# Prefrontal Cortex Response to Prenatal Insult and Postnatal Opioid Exposure

**DOI:** 10.3390/genes13081371

**Published:** 2022-07-30

**Authors:** Haley E. Rymut, Laurie A. Rund, Bruce R. Southey, Rodney W. Johnson, Jonathan V. Sweedler, Sandra L. Rodriguez-Zas

**Affiliations:** 1 Department of Animal Sciences, University of Illinois at Urbana-Champaign, Urbana, IL 61801, USA; hrymut2@illinois.edu (H.E.R.); larund@illinois.edu (L.A.R.); southey@illinois.edu (B.R.S.); rwjohn@illinois.edu (R.W.J.); 2 Neuroscience Program, University of Illinois at Urbana-Champaign, Urbana, IL 61801, USA; jsweedle@illinois.edu; 3 Division of Nutritional Sciences, University of Illinois at Urbana-Champaign, Urbana, IL 61801, USA; 4 Department of Chemistry, University of Illinois at Urbana-Champaign, Urbana, IL 61801, USA; 5 Department of Statistics, University of Illinois at Urbana-Champaign, Urbana, IL 61801, USA

**Keywords:** RNA-seq, opioid, infection, pathways

## Abstract

The influence of proinflammatory challenges, such as maternal immune activation (MIA) or postnatal exposure to drugs of abuse, on brain molecular pathways has been reported. On the other hand, the simultaneous effects of MIA and drugs of abuse have been less studied and sometimes offered inconsistent results. The effects of morphine exposure on a pig model of viral-elicited MIA were characterized in the prefrontal cortex of males and females using RNA-sequencing and gene network analysis. Interacting and main effects of morphine, MIA, and sex were detected in approximately 2000 genes (false discovery rate-adjusted *p*-value < 0.05). Among the enriched molecular categories (false discovery rate-adjusted *p*-value < 0.05 and −1.5 > normalized enrichment score > 1.5) were the cell adhesion molecule pathways associated with inflammation and neuronal development and the long-term depression pathway associated with synaptic strength. Gene networks that integrate gene connectivity and expression profiles displayed the impact of morphine-by-MIA interaction effects on the pathways. The cell adhesion molecules and long-term depression networks presented an antagonistic effect between morphine and MIA. The differential expression between the double-challenged group and the baseline saline-treated Controls was less extreme than the individual challenges. The previous findings advance the knowledge about the effects of prenatal MIA and postnatal morphine exposure on the prefrontal cortex pathways.

## 1. Introduction

Proinflammatory conditions influence brain molecular pathways underlying neurological and behavioral disorders. Maternal immune activation (MIA) during gestation can induce proinflammatory conditions that increase the offspring’s risk for neurodevelopmental disorders, including autism spectrum disorder (ASD) and schizophrenia spectrum disorder (SSD) [1,2,3]. Lipopolysaccharide (LPS) or polyinosinic-polycytidylic acid (Poly(I:C))-induced MIA has been associated with disruptions in the electrophysiological activity of dopaminergic cells and reduction in the number of spontaneously active cells and cell firing rate in the midbrain of rodents [4]. Moreover, the striatum of mice exposed to LPS-elicited MIA presented higher levels of the dopamine 1 receptor (DRD1) [5]. The sensory cortex and hypothalamus of adult male rats exposed to Poly(I:C)-elicited MIA had a persistent increase in the expression of cannabinoid 1 receptor (*CB1*) [6].

The results from the MIA studies offer evidence of the impact of inflammation during gestation on molecular pathways associated with drug abuse. These findings were consistent with the understanding that proinflammatory signals can be modulated by exposure to drugs of abuse. Morphine exposure can disrupt the inflammatory and nervous systems, alter behavior, and exacerbate pronociceptive pain [7,8,9,10].

The connection between the effects of MIA and drugs of abuse at the molecular level supports the high comorbidity between MIA-associated diseases such as ASD and SSD addiction [11,12]. Cannabis exposure during adolescence has been associated with an increased risk of developing SSD later in life and early SSD onset [13,14,15]. The combination of MIA and a second proinflammatory hit after birth, such as exposure to drugs of abuse, increases the likelihood of offspring developing behavioral or psychiatric disorders such as ASD, SSD, anxiety, and depression [13,14,15]. Moreover, significant changes were detected in the entorhinal cortex transcriptome of Poly(I:C)-challenged MIA rats that received cannabinoid injections during adolescence [16]. The previous transcriptome disruptions were driven by genes participating in neurotransmission, SSD, and cellular signaling processes.

Results from the simultaneous study of MIA and postnatal exposure to drugs of abuse have been inconsistent. Poly(I:C)-elicited MIA enhanced the conditioned place preference to amphetamine and amphetamine/cocaine cross-sensitization in male mice, whereas MIA did not impact stereotypical amphetamine-elicited behaviors, including activity, sniffing, licking, and gnawing [11]. In addition, LPS-elicited MIA increased the locomotor-stimulant effect of acute amphetamine exposure and accelerated behavioral sensitization to chronic amphetamine exposure in adult male mice [17]. On the other hand, LPS-elicited MIA did not affect the locomotor behavioral sensitization to chronic amphetamine exposure. Lastly, the administration of cannabinol THC during adolescence attenuated some of the deleterious effects of Poly(I:C)-elicited MIA on the signaling between dopamine cells in the ventral tegmental area of rats [4]. Cannabinoid treatment also reduced behavioral responses to nicotine but did not affect the response to cocaine.

A pig model of virus-elicited MIA constitutes an effective and established system to study effects on brain regions that regulate behaviors [18]. Our previous reports on the effects of MIA followed by the postnatal secondary stress of weaning detected significant changes in the transcriptome leading to enrichment of cocaine, morphine, and nicotine addiction pathways in the pig hippocampus and amygdala [19,20]. We postulate that the porcine MIA model is well-suited to investigate the simultaneous effects of MIA and postnatal exposure to drug abuse. Moreover, studies in pigs and rodents have offered evidence that the effects of MIA and reward-dependency on the behaviors and underlying molecular pathways can be sex-dependent [3,21,22,23].

The present study advances the understanding of the interacting effects between MIA and morphine exposure. The effects of the pre and postnatal hits were characterized in the prefrontal cortex of female and male pigs to enable the identification of MIA-, morphine-, and sex-dependent profiles. A supporting objective of this study is to visualize the impact of MIA and morphine on the gene pathway profiles.

## 2. Materials and Methods

### 2.1. Animal and Sequencing Experiments

All animal experiments were approved by the Illinois Institutional Animal Care and Use Committee (IACUC) at the University of Illinois and followed published protocols [22]. Altogether, 57 pigs born from Camborough gilts inseminated with PIC boars were studied. The pigs and the mothers were housed in individual disease containment chambers, kept on a 12-h light/dark cycle, and fed a complete gestational diet with *ad libitum* access to water. A group of 16 pigs evenly distributed between sexes was born and nursed by gilts not exposed to proinflammatory conditions during gestation, serving as baseline Controls.

A group of 41 pigs were exposed to MIA conditions elicited by maternal nasal inoculation on day 76 of gestation and were nursed by the mothers. The MIA treatment involved inoculation with the porcine respiratory and reproductive virus (strain P129-BV, Purdue University, West Lafayette, IN, USA). The intranasal viral inoculation used a 5 mL of 1 × 10^5^ median tissue culture infectious dose diluted in sterile Dulbecco’s modified Eagle medium [24,25]. Infection was confirmed by significant differences in body temperature and feed intake for 10 days after inoculation between Control and MIA gilts [24]. The MIA gilts were fed the maximum daily, feed refusal was measured daily, and the non-challenged gilts were fed the same amount consumed by MIA gilts during the preceding day [3]. The pigs were born following gilt induction on gestation day 113 using established protocols [22], and were nursed by gilts that received a nutritionally balanced lactation diet. No significant difference in maternal care between both gilt groups was observed and the average weight of the pigs born from both groups was not significantly different (*p*-value > 0.1).

Among the MIA-exposed offspring, a group of 12 pigs was administered morphine to study the simultaneous effect of pre- and postnatal challenges. On postnatal day 17, morphine was administered subcutaneously in the upper thigh once daily at approximately 10:15 AM for four consecutive days at 10 mg/kg bodyweight. The morphine sulfate injection concentrate was administered on postnatal days 17 to 20 (2500 mg/50 mL, Hospira Inc., Lake Forest, IL, USA). The morphine dose and schedule in the present study elicited reward-dependent behavior changes in pigs and rodents [7,9,26,27]. The study of MIA and morphine challenges within and across sexes enabled the detection of sex-dependent and independent effects. An even number of females and males received morphine, and the non-opioid treated MIA, and Control pigs received sterile saline injections matching in volume and schedule that of the matching pigs receiving morphine injections following published protocols [7,9].

Pigs were anesthetized intramuscularly on postnatal day 21 using telazol:ketamine:xylazine (50 mg of tiletamine; 50 mg of zolazepam, and 2.5 mL of xylazine; Fort Dodge Animal Health, Fort Dodge, IA, USA) at a dose of 0.03 mL/kg bodyweight following established protocols. Following anesthesia, an intracardiac injection of sodium pentobarbital (86 mg/kg bodyweight, Fatal Plus, Vortech Pharmaceuticals, Dearborn, MI, USA) was used to euthanize the pigs. The brains were removed, dissected, flash-frozen on dry ice, and stored at −80 °C following published protocols [19]. The EZNA isolation kit (Omega Biotek, Norcross, GA, USA) was used to isolate RNA as previously described [3]. TruSeq Stranded mRNAseq Sample Prep kit (Illumina Inc, San Diego, CA, USA) was used to prepare RNAseq libraries. The libraries were quantitated by qPCR and sequenced on one lane on a NovaSeq 6000 (Illumina Inc, San Diego, CA, USA) for 151 cycles from each end of the fragments using NovaSeq S4 reagent kit [19,20]. The bcl2fastq version 2.20 conversion software was used to produce and demultiplex FASTQ files. The raw and normalized gene expression levels are available in the National Center for Biotechnology Information (NCBI) Gene Expression Omnibus (GEO) database, series identifier GSE209907.

### 2.2. Differential Expression and Network Analysis

The assessment of read quality indicated a minimum Phred score of 35 across all read positions using the FASTQC software [28]. Therefore, no reads required trimming. Paired-end reads were aligned to the *Sus scrofa* genome version 11.1 and quantified using the software kallisto version 0.43.0 [29] with default settings. Genes supported by more than 5 transcripts per million RNA molecules by each morphine-MIA-sex combination were analyzed for differential gene expression using edgeR (v. 3.14.0) in the R version 3.3.1 environment [30]. The normalized gene expression values were described using a generalized linear model encompassing the effects of MIA, morphine treatment, sex, and the two-way interactions. The cut-off for differential expression was set using the false discovery rate (FDR) multiple-testing adjustment [31], encompassing an FDR-adjusted *p*-value < 0.05 and log2 (fold change between treatment groups) > |1.2| to highlight strong trends. A total of 8 (2 × 2 × 2) groups, characterized by the morphine treatment (M = morphine and S = saline), prenatal activation (A = MIA and C = Control), and sex (females and males), were compared. Within sex, the group nomenclature included the morphine treatment followed by the MIA level. Therefore, MA vs. SC describes the comparison between morphine-treated MIA pigs versus saline-treated Control pig.

Positive log2(fold change) estimates indicate over-expression in morphine relative to saline pigs, MIA relative to Control (non-MIA-exposed) pigs, and females relative to males, while negative log2 (fold change) estimates indicate the opposite patterns. For the interactions, a positive log2 (fold change) estimate corresponds to over-expression in double-challenged pigs (i.e., morphine-treated and MIA-exposed) relative to single-challenged pigs (i.e., either morphine or MIA), over-expression in morphine-treated females relative to saline-treated females or morphine-treated males, and over-expression in MIA-exposed females relative to Control females or MIA males

Gene Set Enrichment Analysis (GSEA) using the *S. scrofa* genome as a reference was performed using the WebGESTALT system [32] to identify over-represented Kyoto Encyclopedia of Genes and Genomes (KEGG) human pathways [33] in consideration of the genes most differentially expressed. The statistical significance of the enrichment was determined by calculating the FDR-adjusted *p*-value from 1000 permutations. The normalized enrichment scores (NES) of pathways are a function of the pathway representation among the genes with extreme log2(fold change) adjusted by the average permutation score. The interpretation of the NES sign follows the same criterion as the log2(fold change). A cut-off for differential expression of FDR-adjusted *p*-value 0.05 and NES > 1.5 was considered to highlight enriched pathways. A preliminary review of the results indicated that the enriched pathways among genes presenting interaction effects between sex and the remaining factors did not surpass the threshold of FDR-adjusted *p*-value < 0.05 and NES > 1.5 (for categories). Therefore, this study focuses on the first order interactions and main effects of sex.

Potential changes in the relationship between genes that showed a significant morphine-by-MIA effect and differential expression between morphine and saline-treated pigs across the MIA and Control groups (FDR-adjusted *p*-value < 0.05) were studied by integrating the expression profiles with molecular relationship information available in the BIOGRID database using the STRING application within the Cytoscape version 3.8.1 platform [34]. In the reconstructed networks, the color of the gene reflects the sign of the fold change, where red denotes under-expression and green denotes over-expression in the first relative to the second pig group encompassed in the studied contrast.

The depiction of gene networks encompassing all genes annotated to the KEGG pathway used the default STRING application settings. These settings included the STRING database system representation that ranged from 0 (absent from all the databases) to 1 (present in all the databases) and edge score that indicates the probability supporting the edge gathered from multiple evidence corrected for the probability of randomly observing an interaction (range 0 to 1, score > 0.7 are considered high confidence) [35]. Smaller gene networks were also generated using stricter criteria, including database representation > and edge score > 0.75 thresholds.

## 3. Results

### 3.1. Sequence and Gene Profiles

The minimum RNA integrity number across the samples was 7.3. The sequencing produced an average of 57.2 million paired-end reads per sample. The average number of paired-end reads per pig was consistent between morphine, MIA, and sex groups, and the coefficient of variation was approximately 0.1. The average number of reads mapped per sample was 72%. Together, 16,533 gene expression profiles were analyzed for the effects of morphine, MIA, sex, and their interactions.

At the FDR-adjusted *p*-value < 0.05, 311 genes had a morphine-by-MIA effect, 56 genes had a MIA-by-sex effect, 156 genes had a morphine-by-sex effect, 735 genes had a MIA effect, 611 genes had a morphine effect, and 107 genes had a sex effect. Supplemental Table S1 lists the genes differentially expressed at FDR-adjusted *p*-value < 0.05 and |log2 (fold change between pig groups)| > 1.2.

### 3.2. Effects of Morphine, Maternal Immune Activation, and Sex on the Prefrontal Cortex Pathways

The GSEA analysis identified 14 enriched KEGG pathways (FDR-adjusted *p*-value < 0.05 and a |NES| > 1.5) among the genes that had morphine, MIA, or interaction effects (Table 1). These pathways belonged to metabolism, the immune system, cellular processes, environmental information processing, genetic information processing, and the nervous system categories. Overall, 12 pathways were enriched among the genes with a morphine effect, nine pathways were enriched among genes with a MIA effect, and 11 pathways were enriched among genes with a morphine-by-MIA effect.

The metabolic and digestive pathway categories encompassing genes that had morphine, MIA, or interaction effects include the oxidative phosphorylation pathway (hsa00190), the drug metabolism pathway (hsa00983), and the protein digestion and absorption pathway (hsa04974). These pathways were enriched among genes under-expressed in morphine-treated pigs and in MIA-exposed pigs. The enriched cellular processing categories included the phagosome (hsa04145) and the apoptosis (hsa04210) pathways. These pathways were enriched among genes under-expressed in morphine-treated pigs and over-expressed in MIA-exposed pigs.

The MIA and morphine-associated enriched pathways related to information processing include two categories. Within the genetic information processing category, the ribosome pathway (hsa03010) was enriched among genes under-expressed in morphine-treated and MIA-exposed pigs. Within the environmental information processing category, the cell adhesion molecules pathway (hsa04514) was enriched among genes under-expressed in morphine-treated pigs and over-expressed in MIA-exposed pigs.

The immune system category included the chemokine signaling pathway (hsa04062), the natural killer cell-mediated cytotoxicity pathway (hsa04650), the herpes simplex infection pathway (hsa05168), and the non-alcoholic fatty liver disease (NAFLD) pathway (hsa04932). These pathways were enriched among genes under-expressed in morphine-treated pigs and over-expressed in MIA-exposed pigs.

The enriched nervous system categories, including long-term depression pathway (hsa04730), the nicotine addiction pathway (hsa05033), and the Parkinson’s disease pathway (hsa05012), presented multiple enrichment patterns. The long-term depression and nicotine pathways were enriched among genes over-expressed in pigs either exposed to MIA or morphine. The Parkinson’s disease pathway was enriched among genes under-expressed in morphine-treated pigs.

Table 2 lists KEGG pathways enriched among genes presenting the effect of sex interacting with morphine treatment or MIA. These categories present |NES| > 1.8, although the most enriched categories reached an FDR-adjusted *p*-value < 0.07. The 6 pathways corresponded to the digestive system, metabolism, genetic information processing, and immune system categories. Of the enriched pathways, four pathways were enriched among genes that had a MIA-by-sex effect, and three pathways were enriched among genes that had a morphine-by-sex effect. While no pathway achieved the FDR-adjusted *p*-value < 0.05 enrichment cutoff among genes presenting sex effects irrespective of other factors, the tryptophan and ether lipid metabolism pathways were enriched at FDR-adjusted *p*-value < 0.1 among genes presenting morphine or MIA patterns influenced by sex. In contrast, the remaining pathways were also enriched in association with morphine or MIA, irrespective of sex effects.

The ether lipid metabolism pathway (hsa00565) was enriched among genes under-expressed in morphine-treated females. The tryptophan metabolism pathway (hsa00380), chemokine signaling pathway (hsa04062), and the herpes simplex infection pathway (hsa05168) were enriched among genes over-expressed in MIA-exposed females. The ribosome pathway (hsa03010) was enriched among genes under-expressed in MIA-exposed females and over-expressed in morphine-treated females.

### 3.3. Effects of Morphine, Maternal Immune Activation, and Sex on the Prefrontal Cortex Gene Expression Profiles

The list of the genes presenting a significant MIA, morphine, sex, or interaction effect (FDR-adjusted *p*-value < 0.05 and |log2 (fold change)| > 1.2), irrespective of the pathway, is summarized in Supplemental Table S1. Genes annotated to the enriched pathways reported in Table 1 and Table 2 are presented in Table 3 and Table 4, respectively.

While considering all significant genes (FDR-adjusted *p*-value < 0.05 and |log2(fold change)| > 1.2), approximately 94% of the genes affected by MIA were over-expressed in MIA relative to Control pigs. The prominent gene families altered by MIA included four genes in the intercrine β (chemokine CC) family, 11 genes in the cluster of differentiation (CD) families, three genes in the C-X-C motif chemokine ligand (CXCL) family, and nine genes in the Swine Leukocyte Antigen (SLA) family.

It is noteworthy that all the genes exhibiting morphine effects were under-expressed in morphine-relative to saline-treated pigs, while approximately 67% of the genes presenting sex effects were under-expressed in females relative to males. Among the genes presenting interaction effects, 56% of the profiles impacted by the morphine-by-MIA interaction were under-expressed in pigs exposed to morphine or MIA relative to other groups, and 60% of the genes presenting morphine-by-sex interaction effects were over-expressed in morphine-treated females relative to other morphine or sex groups. Lastly, approximately 91% of the genes showing MIA-by-sex interaction patterns were over-expressed in MIA-exposed female pigs relative to other MIA or sex groups.

### 3.4. Effects of Morphine, Maternal Immune Activation and Sex on Prefrontal Cortex Networks

**Figure 1:**
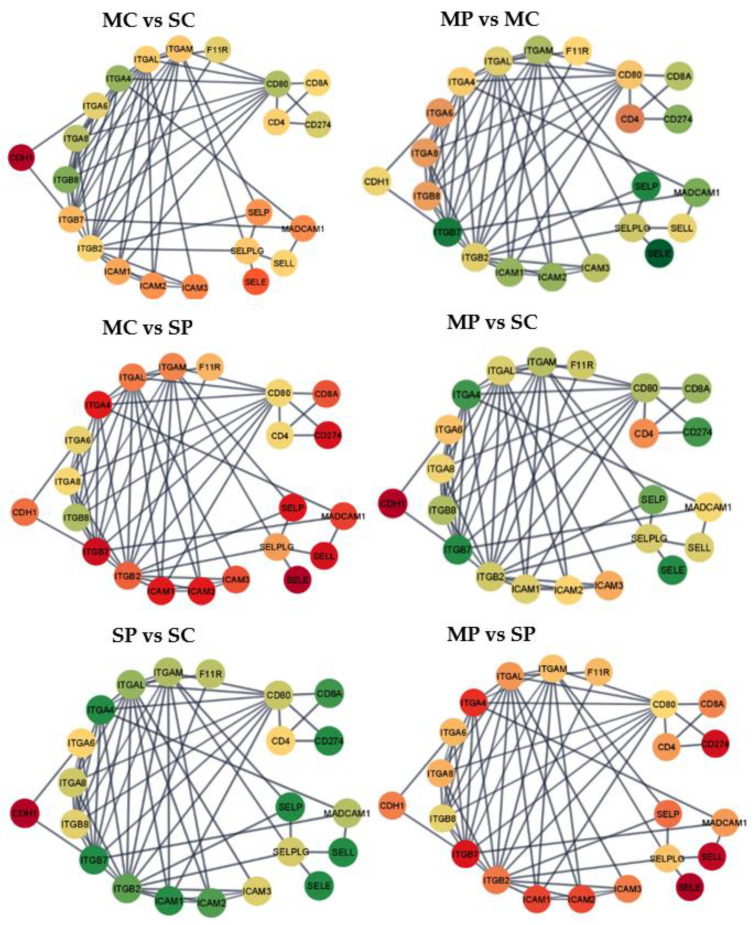
Gene networks for the cell adhesion molecules pathway. Nodes = genes; edges = STRING database connections; node color = differential expression between groups characterized by opioid (M = morphine or S = saline) followed by MIA exposure (C = control or P = viral MIA) from red (under-expressed in the first vs. second group, to yellow, and green (over-expressed in the first versus second group. Networks: MC vs. SC = morphine-treated Control vs. saline-treated Control; MP vs. MC = morphine-treated MIA vs. morphine-treated Control; MC vs. SP = morphine-treated Control vs. saline-treated MIA; MP vs. SC = morphine-treated MIA vs. saline-treated Control; SP vs. SC = saline-treated MIA vs. saline-treated Control; and MP vs. SP = morphine-treated MIA vs. saline-treated MIA.

**Figure 2:**
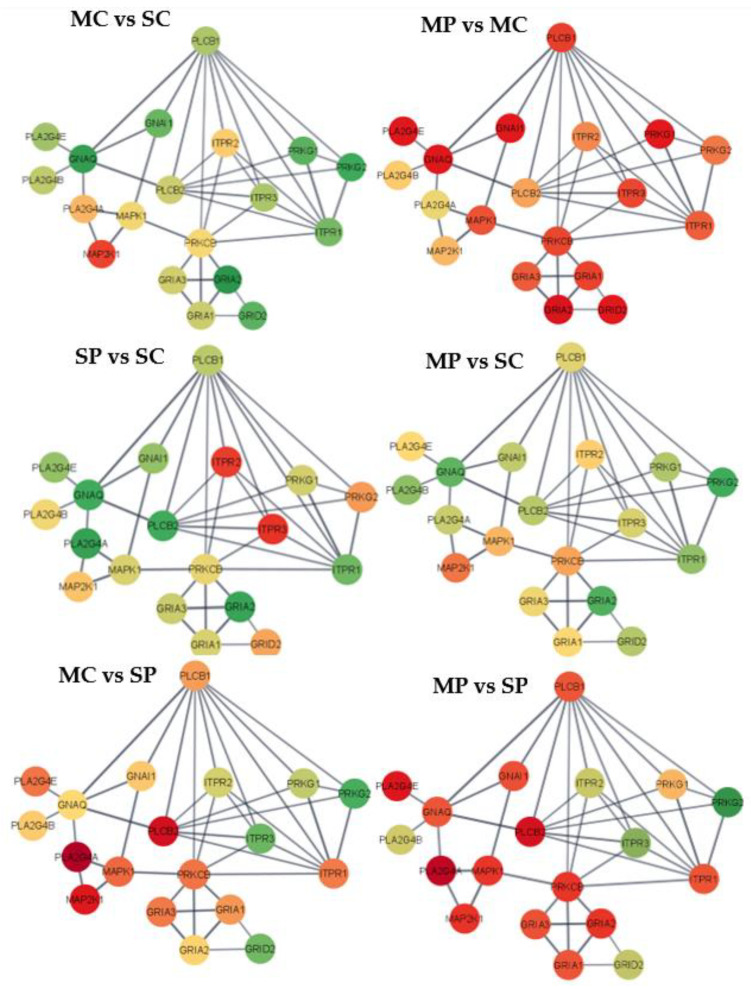
Gene networks for the long-term depression pathway. Nodes = genes; edges = STRING database connections; node color = differential expression between groups characterized by opioid (M = morphine or S = saline) followed by MIA exposure (C = control or P = viral MIA) from red (under-expressed in the first vs. second group, to yellow, and green (over-expressed in the first versus second group. Networks: MC vs. -SC = morphine-treated Control vs. saline-treated Control; MP-MC = morphine-treated MIA vs. morphine-treated Control; MC vs. SP = morphine-treated Control vs. saline-treated MIA; MP vs. SC = morphine-treated MIA vs. saline-treated Control; SP vs. SC = saline-treated MIA vs. saline-treated Control; and MP vs. SP = morphine-treated MIA vs. saline-treated MIA.

To complement the pathway enrichment and gene expression patterns, the study of gene interactions and differential expression provided insights into the potential impact of MIA and morphine treatment on gene relationships. Based on enrichment levels and the predominance of differential expression, the cell adhesion molecules pathway and the long-term depression pathway were recreated. Condensed circle layout networks, including 22 genes in the adhesion molecules pathway (Figure 1) and 19 genes in the long-term depression pathway (Figure 2), highlight the impact of the factors studied. The entire networks of 42 genes in the cell adhesion molecules pathway and 29 genes in the long-term depression pathway are depicted in Supplemental Figures S1 and S2.

The six group contrasts depicted in the networks correspond to, MC-SC = morphine-treated Control group vs. saline-treated Control group; MP-MC = morphine-treated MIA vs. morphine-treated Control group; MC-SP = morphine-treated Control vs. saline-treated MIA; MP-SC = morphine-treated MIA vs. saline-treated Control group; SP-SC = saline-treated MIA vs. saline-treated Control group; and MP-SP = morphine-treated MIA vs. saline-treated MIA. The node colors range from red corresponding to genes with negative log2 (fold change) between the first and second treatment group and green corresponding to a positive log2 (fold change). Yellow nodes correspond to genes presenting the median fold change among genes and treatment group comparisons, while the red and green range was capped at the 10th and 90th percentile of differential expression across genes and treatment groups to optimize visualization and limit spectrum extremes corresponding to outlier gene profiles. The log2 (fold changes) for each gene and contrast in the cell adhesion molecules pathway and in the long-term depression pathway are listed in Supplemental Tables S2 and S3.

Among the 42 genes in the cell adhesion molecules pathway, 25 genes were over-expressed in morphine relative to saline-treated Control pigs (Figure 1 MC vs. SC), while only five are over-expressed in MIA-exposed pigs (Figure 1 MP vs. SP; Supplemental Table S2). The most extreme over-expression in morphine relative to saline-treated Controls was observed for the genes cluster of differentiation 80 (*CD80*), cadherin 2 (*CDH2*), contactin 1 (*CNTN1*), integrin α-6 (*ITGA6*), integrin subunit α 8 (*ITGA8*), integrin β-8 (*ITGB8*), neural cell adhesion molecule 2 (*NCAM2*), neuronal growth regulator 1 (*NEGR1*), neurofascin (*NFASC*), neuroligin-1 (*NLGN1*), neuron-glia related cell adhesion molecule (*NRCAM*), and versican (*VCAN*). The most extreme under-expression in morphine relative to saline-treated Controls was observed for L1 cell adhesion molecule protein (*L1CAM*), *NEGR1*, *NEGR1*, neurexin-3-α (*NRXN3*), netrin G1 (*NTNG1*), and *SDC1*.

Overall, 40 genes were over-expressed in saline-treated MIA relative to Controls (Figure 1 SP vs. SC) and 26 genes were over-expressed in morphine-treated MIA relative to Control groups (Figure 1 MP vs. MC). The most over-expressed genes in saline-treated MIA relative Controls were calcium-dependent adhesion protein or cadherin 1 (*CDH1*), *CLDN6*, intercellular adhesion molecule 3 (*ICAM3*), integrin α M chain (*ITGAM*), mucosal addressin cell adhesion molecule (*MADCAM*), and P-selectin glycoprotein ligand-1 (*SELPLG*). The most under-expressed genes were in the CD family including membrane glycoprotein of T lymphocytes CD4 (*CD4*), activation antigen of T-lymphocyte CD80 (*CD80)*, *CDH2*, *CNTN1*, *ITGA6*, *ITGA8*, *ITGB8*, *NCAM2*, *NEGR1*, *NFASC*, *NLGN1*, *NRCAM*, and *VCAN*. On the other hand, the genes most differentially expressed between morphine-treated MIA and Control groups included *CD2*, *CD22*, *CD274*, tumor necrosis factor receptor superfamily member 5 (*CD40*), T-cell differentiation antigen (*CD6*), *CD8A*, F11 receptor (*F11R*), intercellular adhesion molecule 1 (*ICAM1*), intercellular adhesion molecule 2 (*ICAM2*), integrin subunit α 4 (*ITGA4*), integrin α L chain (*ITGAL*), integrin subunit β 2 (*ITGB2*), integrin subunit β 7 (*ITGB7*), *L1CAM*, *NRXN3*, *NTNG1*, protein tyrosine phosphatase receptor type C (*PTPRC*), syndecan-1 (*SDC1*), selectin E (*SELE*), selectin L (*SELL*), selectin P (*SELP*), sialic acid binging Ig like lectin 1 (*SIGLEC1*), sialophorin (*SPN*), and T cell immunoreceptor (*TIGIT*).

Figure 1 MC vs. SP depicts the interactions between the effects of morphine and MIA on the prefrontal cortex expression of genes in the cell adhesion molecules pathway. The contrast between morphine-treated Control and saline-treated MIA pigs presented the most extreme expression differential among all contrasts (Supplemental Table S2). The most extreme over-expression in morphine-treated Control relative to saline-treated MIA pigs was detected in CD4, while under-expression was detected in *CD2*, *CD22*, *CD274*, *CD40*, *CD6*, *CD8A*, *F11R*, *ICAM1*, *ICAM2*, *ICAM3*, *ITGA4*, *ITGAL*, *ITGAM*, *ITGB2*, *ITGB7*, *MADCAM*, *PTPRC*, *SDC1*, *SELE*, *SELL*, *SELP*, *SELPLG*, *SIGLEC1*, *SPN*, and *TIGIT*. Conversely, the positive log-fold change of *CD80* stands out in the comparison between morphine-treated MIA and saline-treated Control pigs.

The behavior of the 29-gene networks for the long-term depression pathway on the prefrontal cortex across the six contrasts furthered the understanding of the individual and interacting effects of morphine and MIA (Figure 2 and Supplemental Table S3). The overarching effects of individual challenges of morphine and MIA in the long-term depression pathway were aligned with the cell adhesion molecules pathway. For the contrast between morphine and saline-treated Control pigs, positive fold change was detected in 28 genes (Figure 2 MC vs. SC). The genes with the highest over-expression in morphine relative to saline-treated Control pigs included B-Raf coding protein (*BRAF*), calcium voltage-gated channel subunit α 1 A (*CACNA1A*), G protein subunit α 13 (*GNA13*), guanine nucleotide-binding protein G(i) α-1 subunit (*GNAI1*), G protein subunit α q (*GNAQ*), glutamate ionotropic receptor AMPA type subunit 1 (*GRIA1*), glutamate ionotropic receptor AMPA type subunit 2 (*GRIA2*), glutamate ionotropic receptor delta type subunit 2 (*GRID2*), guanylate cyclase 1 soluble subunit α 2 (*GUCY1A2*), insulin like growth factor 1 receptor (*IGF1R*), Kirsten rat sarcoma viral oncogene homolog (*KRAS*), phospholipase C β 1 (*PLCB1*), protein phosphatase 1 regulatory subunit 17 (*PPP1R17*), protein kinase cGMP-dependent 1 (*PRKG1*), and protein kinase cGMP-dependent 2 (*PRKG2*). The most extreme negative log-fold changes between morphine and saline-treated MIA pigs were detected in glutamate ionotropic receptor AMPA type subunit 3 (*GRIA3)*, glutamate metabotropic receptor 1 (*GRM1*), inositol 1,4,5-triphosphate receptor type 1 (*ITPR1*), tyrosine-protein kinase (*LYN*), mitogen-activated protein kinase 1 (*MAPK1*), phospholipase A2 group IVE (*PLA2G4E*), and protein kinase C β (*PRKCB*). Interestingly, the highest percentage of genes presenting positive log-fold change and amounting to 52% of the genes in the long-term depression pathway was observed in morphine relative to saline-treated Control pigs.

The contrast between MIA and Control saline-treated pigs encompassed nine genes over-expressed in MIA-exposed pigs (Figure 2 MP vs. SP). The genes with an extreme positive log-fold change between MIA and Control saline-treated pigs included *GRIA3*, *GRM1*, *ITPR1*, *LYN*, mitogen-activated protein kinase kinase 1 (*MAP2K1*), *MAPK1*, phospholipase A2 group IVA (*PLA2G4A*), *PLA2G4E*, phospholipase C β 2 (*PLCB2*), and *PRKCB*. The genes with the most extreme under-expression in morphine-treated MIA relative to Control pigs included *BRAF*, *CACNA1A*, *GNA13*, *GNAI1*, *GNAQ*, *GRIA1*, *GRIA2*, *GRID2*, *GUCY1A2*, *IGF1R*, *KRAS*, phospholipase A2 group IVB (P*LA2G4B*), *PLCB1*, *PPP1R17*, *PRKG1*, and *PRKG2*.

The most over-expressed genes in morphine-treated Control relative to saline-treated MIA pigs included inositol 1,4,5-triphosphate receptor type 2 (*ITPR2*) and inositol 1,4,5-triphosphate receptor type 3 (*ITPR3*). For the same contrast, the most under-expressed genes included *MAP2K1*, *PLA2G4A*, *PLA2G4B*, *PLCB2*, and ryanodine receptor (*RYR1*). The comparison between morphine-treated MIA and saline-treated Controls had the highest number of under-expressed genes amounting to 55% of genes in the long-term depression pathway (Supplemental Table S2). The genes *PLA2G4B* and *RYR1* were among the genes with extreme positive log-fold change between the double hit morphine-treated MIA and saline-treated Control pigs.

## 4. Discussion

Research in biomedical models, including rodents and pigs, has advanced the behavioral and molecular characterization of the effect of postnatal repeated exposure to drugs of abuse and MIA. The morphine dose (10 mg/kg) and schedule (17 to 20 days of age) in the present study elicited reward-dependent behavior changes in pigs and rodents [7,9,26,27]. Pigs subcutaneously injected with 10 mg/kg of morphine presented no or significantly reduced cutaneous trunci response to pin-prick stimulation up to 10 h after injection, and normal response resumed afterward [26]. In addition to changes in nociception response, the rate of locomotor activity was significantly greater in morphine-treated pigs. Mice that were subcutaneously injected 10 mg/kg of morphine sulfate on four alternating days had higher hot plate antinociception, lower formalin-induced pain behavior, and developed conditioned place preference [27]. The effect of morphine injection in mice at a dose (10 mg/kg) and schedule (four days prior to sampling) similar to those in the present study included opioid-induced hyperalgesia [7,9]. Behavioral changes have also been observed in the pig MIA model used in the present study, including a reduction in social behavior and changes in locomotor behaviors [18,24].

The effects of the proinflammatory agents MIA and morphine on the brain molecular pathways that underlie behavioral changes have been reported. However, the simultaneous impact of MIA and drugs of abuse remains partially understood. Our results on the prefrontal cortex of female and male pigs exposed to viral-induced MIA during gestation and repeatedly exposed to morphine provided insights into interacting gene expression patterns.

### 4.1. Prefrontal Cortex Genes and Pathways Influenced by Morphine, Maternal Immune Activation, and Sex

Among the factors studied, approximately 2000 genes were differentially expressed (FDR-adjusted *p*-value < 0.05), and the effects of MIA and morphine were more prevalent than the effects of sex as main effects or interactions. The effect of MIA alone or interacting with morphine treatment or sex was detected in 1102 genes, whereas the effect of morphine alone or interacting with MIA or sex was detected in 1078 genes, and sex effects were detected in 319 genes.

Functional analysis of the differentially expressed genes offered insights into the pathways impacted by MIA and morphine (Table 1). Main pathways enriched among genes presenting significant morphine-by-MIA interaction effects included oxidative phosphorylation, ribosome, chemokine signaling, cell adhesion molecules, and long-term depression. The interaction effect is characterized by relative changes in the differential expression between two levels of one factor across the levels of the other factor. Reports of differential expression associated with MIA or morphine effects support the simultaneous changes across MIA and morphine levels observed in the present study.

The significant and negative enrichment score of the oxidative phosphorylation pathway associated with morphine effects and significant interaction were characterized by underexpression in the morphine-treated MIA group relative to the saline-treated Control baseline group. The previous pattern is consistent with reports that several proteins involved in oxidative phosphorylation were downregulated in the prefrontal cortex of rats after repeated morphine exposure [36]. Similar interaction and main effect profiles were observed in the enriched ribosome pathway, where genes were under-expressed in morphine- relative to saline-treated pigs. The ribosome pathway profile is comparable to the under-expression of ribosomal genes in the prefrontal cortex of amphetamine-treated mice [37]. The ribosome pathway was also enriched among differentially expressed in the striatum of a mouse line selected for reward dependency for increased voluntary wheel-running behavior relative to a control line [38].

Aligned with our results on morphine effects, more than 500 genes were differentially expressed in the prefrontal cortex of rats after repeated morphine injections, and 35% of these profiles were detected in both sexes [23]. Likewise, the enrichment of the pathways of long-term depression (associated with synaptic strength) and signaling cell adhesion molecules (associated with inflammation and neuronal development) that were detected in the present analysis is consistent with the enrichment of synaptic long-term depression, signaling, and plasticity pathways detected in the prefrontal cortex of morphine-treated rats [23]. The observed interaction effects on the previous two pathways were characterized by gene under-expression in the morphine-treated group or over-expression in the MIA group relative to the corresponding baselines. This effect is consistent with the reported expression changes in the hippocampus of rats exposed to LPS-induced MIA [39]. The consistency between the MIA and morphine effects detected in the present study and those reported for other brain regions may be related to the prefrontal cortex neurons that project into the amygdala and forward to the hippocampus and back to the prefrontal cortex [40]. Moreover, the role of cell adhesion molecules in the context of MIA and ASD has been reviewed and synaptic cell adhesion molecules are well-characterized ASD risk genes [41].

The enrichment of the protein digestion and absorption category among genes impacted by interaction effects and under-expressed in the morphine-treated MIA group aligns with the same category enrichment among metabolic alterations in the prefrontal cortex of 15 patients with schizophrenia relative to controls [42]. Likewise, an analysis of the frontal cortex from 24 individuals with schizophrenia and 25 unaffected controls identified 1146 genes differentially expressed between groups [43]. The interaction effect associated with the enrichment of immune categories encompassed gene over-expression in the MIA or under-expression in the morphine-treated group when compared with Control and saline-treated groups. The enrichment of immune categories (e.g., chemokine signaling, Herpes simplex infection) can be related to the enrichment of inflammatory pathways among genes differentially expressed in the prefrontal cortex of individuals with schizophrenia relative to controls [43]. The over-representation of the ribosome pathway among genes presenting an interaction effect including under-expression in morphine-treated MIA pigs in the prefrontal cortex is consistent with alternative splicing and gene profiles in the amygdala of pigs exposed to weaning stress and MIA [19,44]. The previous enrichment and pattern are consistent with the under-expression of genes in the ribosome pathway detected in fetal mouse brain exposed to Poly (I:C)-elicited MIA relative to control mice [45].

The most enriched categories among the genes presenting MIA-by-sex effects were the tryptophan metabolism and the ribosome pathways, albeit at lower significance levels than the morphine-by-MIA interaction. The inflammatory signals can modulate the enzymatic activity in the tryptophan pathway favoring the metabolization of tryptophan [46], and our results are aligned with reports of hindered kynurenine pathway metabolism in the prefrontal cortex of SSD-diagnosed individuals [47]. Changes in the tryptophan pathway have been associated with decreased hyperinflammation, increased long-term immune tolerance [48], and depression [49]. In addition, ribosomes contribute to innate immune response [50,51]. Understanding these relationships is important to address sex differences in therapies associated with inflammation.

In the present study, the pathways of cell adhesion molecules and long-term depression were enriched among genes presenting significant interaction between morphine and MIA effects. However, while the cell adhesion molecules pathway was enriched for the respective effects of MIA and morphine, the long-term depression pathway was enriched among genes presenting interaction effects. The complementary profiles of the previous pathways prompted the investigation of the corresponding gene networks to gain additional insights into the range of responses to MIA and drugs of abuse previously reported.

### 4.2. Interacting Effects of Morphine Exposure and Maternal Immune Activation on the Cell Adhesion Molecule Pathway in the Prefrontal Cortex

The most common expression pattern among the genes in the cell adhesion molecule pathway was over-expression in saline-treated MIA relative to saline-treated Control and morphine-treated MIA groups. The second most common expression pattern was a subset of the previous pattern, with genes over-expressed in the morphine-treated MIA relative to the morphine-treated Control group. In the absence of MIA, the effect of morphine was mild for many genes in the pathway. In the cell adhesion molecule pathway, MIA challenge elicited gene over-expression under saline conditions, whereas morphine treatment elicited gene under-expression under MIA conditions. A proteomic study of presynaptic proteins reported downregulation of cell adhesion proteins (including *NCAM1*) in rats repeatedly exposed to morphine for five days [52]. While MIA appears to prime the expression of genes in the cell adhesion molecules pathway, morphine appears to revert the previous effect. While the individual effects of morphine and MIA detected in the prefrontal cortex are consistent with previous reports, the antagonistic effects of simultaneous morphine and MIA exposure may relate to reports that cannabinoids may ameliorate the symptoms of individuals with MIA-related disorders [16].

The effects of MIA and morphine detected in the present study are aligned with profiles from studies focused on studying MIA or morphine. Concerning the effects of morphine irrespective of MIA, the cell adhesion molecules pathway was enriched among genes under-expressed in the morphine-treated group relative to saline-treated pigs. Our findings are aligned with reports that cell adhesion molecules participate in neuroadaptation processes triggered by reward dependency and drugs of abuse [53]. Consistent with the patterns identified in the present study, lower levels of cell adhesion molecules were correlated with increased morphine analgesia [54]. Morphine dependence was also associated with a significant decrease in the polysialylated form of neural cell adhesion molecule in the adult rat hippocampus [55].

Among genes in the cell adhesion molecules pathway impacted by morphine, *PTPRC* was under-expressed in morphine-treated pigs. Similarly, the tyrosine phosphatase receptor type Z (*PTPRZ1*) was downregulated by chronic morphine treatment and upregulated after acute treatment in rodents [56]. Gene *CDH1* participates in neurogenesis and cortical development and was under-expressed in the morphine-treated relative to the saline group (Table 2). This pattern is aligned with reports of reduced expression in the brainstems of morphine-exposed female mice [57].

Regarding the effect of MIA irrespective of morphine treatment, the cell adhesion molecules pathway was enriched among genes over-expressed in the MIA-exposed group relative to the Control group. The over-expression pattern is consistent with reports of increased levels of many cerebral cell adhesion molecules in LPS-elicited MIA mice that were subsequently challenged with LPS in adulthood [58]. Moreover, the pathological features of the MIA-related conditions ASD and SSD have been associated with dysfunction in synapse excitation/inhibitory balance and synaptic transmission and trans-synaptic recognition and signaling processes that are mediated by cell adhesion molecules [59,60].

Several genes and gene families in the cell adhesion molecules pathway that presented differential expression across MIA groups have been previously associated with a prenatal immune challenge (Table 2). Consistent with our findings on selectin genes, including *SELP*, *SELL*, and *SELE*, MIA disrupted the expression of genes *SELP* and *SELL* in mice brains [61]. Moreover, consistent with the detected patterns of multiple integrins including *ITGB2*, *ITGA4*, *ITGB7*, and *ITGAL*, detected in the present study, the expression of the neurodevelopmental gene *ITGB2* was altered in the brain of individuals diagnosed with SSD or ASD [62]. Likewise, high levels of ICAM1 were reported in the dorsolateral prefrontal cortex of individuals with SSD [63]. Lastly, the MIA-associated changes in the expression changes in CD genes are in agreement with reports that *CD40* was activated in the brain of SSD patients [64] and that *CD6* was over-expressed in the brain of individuals with ASD [65].

### 4.3. Interacting Effects of Morphine Exposure and Maternal Immune Activation on the Long-Term Depression Pathway in the Prefrontal Cortex

In the long-term depression pathway, the most common expression pattern is characterized by gene over-expression in morphine-treated Control and saline-treated MIA relative to the saline-treated Control group. The second most common expression pattern is a subset of the previous pattern, with gene under-expression in morphine-treated MIA relative to morphine-treated Control and saline-treated groups. Therefore, the overall pattern is characterized by over-expression in the morphine-treated Control relative to the MIA and saline-treated Control groups having the lowest expression levels.

The over-expression of long-term depression genes in morphine-treated Control and saline-treated MIA relative to the double challenge and no challenge groups is congruous with published work that evaluated the factors separately. Concerning morphine effects, the long-term depression system is known to be involved in the pathological states of drug addiction [66]. Long-term depression pathway genes were over-expressed in the prefrontal cortex of rats repeatedly exposed to morphine relative to controls [23], and repeated exposure to stimulant drugs of abuse induced a long-term depression-like state at glutamatergic synapses in the prefrontal cortex of mice [67]. Drugs of abuse, including morphine, are known to modulate synaptic long-term depression and long-term potentiation processes in the nucleus accumbens [68]. The enrichment of the long-term depression pathway among gene profiles impacted by MIA is aligned with reports that cortical neurons from rats exposed to Poly (I:C)-elicited MIA had increased levels of MHC class I genes, accompanied by long-term depression enrichment, lower synapse density, and impairments in neural connectivity [69].

Phospholipases A2 enzyme genes, including *PLA2G4B*, *PLA2G4A*, and *PLA2G4E*, were among the long-term depression gene families presenting differential expression associated with MIA (Table 2). Activation of phospholipases enhances the production of prostaglandin E2, which plays an anti-inflammatory role in the brain [70], consistent with the over-expression in MIA relative to Control pigs. Moreover, *PLA2GA* was over-expressed in the peripheral blood mononuclear cells in SSD compared to control individuals [71], and genetic variants of this family have been associated with depression [72]. Furthermore, also impacted by MIA were genes in the protein kinase cGMP-dependent family, including *PRKG1*, *PRKG2*, and *PRKCB* (Table 2). Enrichment of the cGMP-dependent PRKG signaling pathway was detected among differentially spliced genes in the amygdala of pigs exposed to MIA [44]. In addition, increased phosphorylation of PRKG targets was reported in the anterior cingulate cortex of SSD compared to control individuals, and PRKG molecule profiles had been linked to ASD [73].

The present study detected expression changes in glutamate receptor genes within the long-term depression pathway that have been associated with behavioral comorbidities of MIA-associated disorders. These glutamate receptors included *GRIA*, *GRID*, *GRIA1*, *GRIA2*, *GRIA3*, *GRID2*, and *GRM1*. Glutamate receptors have been associated with increased anxiety behaviors observed in mice exposed to an inflammatory hypercaloric diet during gestation and after birth [74]. Likewise, the profiles of genes in the inositol 1,4,5-trisphosphate receptor family, including *ITPR1*, *ITPR2*, and *ITPR3*, were impacted by MIA, and members of this family have been associated with neurological and motor function impairment and bipolar disorder [75].

## 5. Conclusions

The present study advanced the understanding of the complex interaction between MIA and morphine on molecular mechanisms in the prefrontal cortex. Morphine and MIA had synergistic effects on the metabolism, protein digestion, and ribosome pathways, whereas antagonistic effects were detected in environment information processing and nervous system pathways. Morphine exposure was associated with the under-expression of genes in predominantly impacted pathways, while MIA induced over- or under-expression, resulting in antagonistic or synergistic interactions among the enriched pathways, respectively.

The cell adhesion molecules and long-term depression pathways shared an antagonistic effect of morphine and MIA on gene expression patterns. The previous effect was evidenced by the moderate differential expression between the double-challenged group (morphine-treated MIA-exposed) and the baseline saline-treated Controls. The range of interacting effects on the prefrontal cortex pathways uncovered in the present study offers insights into the interplay between the effects of MIA and morphine.

## Figures and Tables

**Table 1:** Enriched pathways (Gene Set Enrichment Analysis False Discovery Rate-adjusted *p*-value < 0.05 and normalized enrichment score absolute value |NES| > 1.5) among genes presenting morphine (M), maternal immune activation (A), or interactions (MxA) effects in the prefrontal cortex.

	KEGG	MxA ^2^		M		A	
Pathway Type and Name	Identifier ^1^	N ^3^	FDR	N	FDR	N	FDR
*Metabolism* ^4^							
Oxidative phosphorylation	hsa00190	2.7	1 × 10^−5^	−2.5	1 × 10^−5^	−2.1	3 × 10^−3^
Drug metabolism	hsa00983			−1.8	2 × 10^−2^		
*Digestive system*							
Protein digestion & absorption	hsa04974	2.3	3 × 10^−2^	−1.9	6 × 10^−3^	−2.2	1 × 10^−3^
*Genetic information processing*							
Ribosome	hsa03010	3.0	1 × 10^−5^	−2.1	2 × 10^−3^	−2.3	1 × 10^−3^
*Immune system*							
Chemokine signaling	hsa04062	−2.0	4 × 10^−3^	−2.0	5 × 10^−3^	2.2	1 × 10^−5^
Natural killer cell med. cytotoxic.	hsa04650	−1.7	3 × 10^−2^	−2.0	5 × 10^−3^	2.1	1 × 10^−5^
*Diseases*							
Herpes simplex infection	hsa05168	−2.2	2 × 10^−4^	−2.1	2 × 10^−3^	2.2	1 × 10^−5^
Non-alcoholic fatty liver disease	hsa04932			−2.1	2 × 10^−3^		
*Cellular Processes*							
Phagosome	hsa04145	−1.7	4 × 10^−2^	−2.3	3 × 10^−4^	2.1	1 × 10^−5^
Apoptosis	hsa04210			−2.0	4 × 10^−3^	1.8	1× 10^−2^
*Environment information processing*							
Cell adhesion molecules	hsa04514	−2.0	3 × 10^−3^	−2.1	3 × 10^−3^	2.0	1 × 10^−4^
*Nervous system*							
Long-term depression	hsa04730	−1.7	5 × 10^−2^				
Nicotine addiction	hsa05033	−1.8	1 × 10^−2^				
Parkinson disease	hsa05012	2.3	5 × 10^−2^	−2.3	1 × 10^−5^		

^1^ KEGG = Kyoto Encyclopedia of Genes and Genomes human pathway identifier. ^2^ MxA = morphine-by-maternal immune activation (MIA) interaction effect where NES > 0 (NES < 0) denotes gene over-expression (under-expression) in morphine-treated MIA-exposed pigs; M = morphine effect where NES > 0 (NES < 0) denotes gene over-expression (under-expression) in morphine- relative to saline-treated pigs; and A = MIA effect where NES > 0 (NES < 0) denotes gene over-expression (under-expression) in MIA relative to Control pigs. ^3^ N = normalized enrichment score (NES); FDR = FDR-adjusted *p*-value. FDR-adjusted *p*-value < 1 × 10^−5^ = 1 × 10^−5^. ^4^ Names in italics denote the pathway type.

**Table 2:** Enriched pathways (Gene Set Enrichment Analysis False Discovery Rate-adjusted *p*-value < 0.1 and normalized enrichment score absolute value |NES| > 1.5) among genes profiles presenting effects of sex (S) alone or interacting with morphine (M) or maternal immune activation (A) in the prefrontal cortex.

	KEGG	AxS ^2^		MxS	
Pathway Type and Name	Identifier ^1^	N ^3^	FDR	N	FDR
*Metabolism* ^4^					
Tryptophan metabolism	hsa00380	1.8	6 × 10^−2^		
Ether lipid metabolism	hsa00565			−1.8	1 × 10^−1^
*Genetic information processing*					
Ribosome	hsa03010	−2.1	7 × 10^−2^	1.9	1 × 10^−1^
*Immune system*					
Chemokine signaling	hsa04062	2.0	6 × 10^−2^		
Herpes simplex infection	hsa05168	1.8	6 × 10^−2^		

^1^ KEGG = Kyoto Encyclopedia of Genes and Genomes human pathway identifier. ^2^ AxS = MIA-by-sex interaction effect where NES > 0 (NES < 0) denotes gene over-expression (under-expression) in MIA females; MxS = morphine-by-sex interaction effect where NES > 0 (NES < 0) denotes gene over-expression (under-expression) in morphine- relative to saline-treated females. No enrichment was detected among genes with sex effects. ^3^ N = normalized enrichment score (NES); FDR = FDR-adjusted *p*-value; FDR-adjusted *p*-value < 1 × 10^−5^ = 1 × 10^−5^. ^4^ Names in italics denote the pathway type.

**Table 3:** Log2(fold change) and False Discovery Rate-adjusted *p*-value (LogFC and FDR, respectively) of the genes in the prefrontal cortex that presented a significant (FDR < 0.05) interaction or main effect of morphine (M) and maternal immune activation (A) and represent the pathways enriched at FDR < 0.05 and a normalized enrichment score |NES| > 1.5.

Gene	MxA ^1^	M		A	
Symbol	FDR	LogFC	FDR ^2^	LogFC	FDR
*Metabolism*					
*TCIRG1*				−0.4	2 × 10^−2^
*AOX1*	5 × 10^−3^				
*FMO1*	1 × 10^−5^			0.5	2 × 10^−3^
*Immune System*					
*CCL2*		−1.4	1 × 10^−5^	2.1	1 × 10^−5^
*CCL4*		−1.4	1 × 10^−5^	1.7	1 × 10^−5^
*CCL5*	2 × 10^−2^	−1.1	4 × 10^−5^	2.9	1 × 10^−5^
*CCR2*				2.1	1 × 10^−5^
*CD48*				1.7	1 × 10^−5^
*CD74*				2.6	1 × 10^−5^
*CXCL11*				4.9	1 × 10^−5^
*NFKBIA*	5 × 10^−2^			0.5	5 × 10^−3^
*CXCL9*				5.5	1 × 10^−5^
*Cellular Processes*					
*CTSS*	5 × 10^−2^	−0.6	8 × 10^−5^	1.4	1 × 10^−5^
*MARCO*		−1.1	2 × 10^−2^	5.6	1 × 10^−5^
*CTSW*				2.3	1 × 10^−5^
*Environment information processing*					
*CD274*	1 × 10^−6^	−0.6	2 × 10^−3^	1.4	1 × 10^−5^
*CD6*				1.9	1 × 10^−5^
*CDH1*	1 × 10^−6^	−1.1	1 × 10^−5^	−0.6	8 × 10^−5^
*Nervous system*					
*RYR1*		0.4	3 × 10^−2^	0.5	9 × 10^−3^

^1^ MxA = morphine-by-maternal immune activation (MIA) interaction effect; M = morphine effect where LogFC > 0 (LogFC < 0) denotes gene over-expression (under-expression) in morphine- relative to saline-treated pigs; and A = MIA effect where LogFC > 0 (LogFC < 0) denotes gene over-expression (under-expression) in MIA-exposed relative to Control pigs. ^2^ FDR-adjusted *p*-value < 1 × 10^−10^; FDR-adjusted *p*-value < 1 × 10^−5^ = 1 × 10^−5^.

**Table 4:** Log2(fold change) and False Discovery Rate-adjusted *p*-value (logFC and FDR, respectively) of genes in the prefrontal cortex that presented a significant (FDR < 0.05) main or interaction effect of sex (S) with morphine (M) and maternal immune activation (A) and represent the pathways enriched at FDR < 0.05 and a normalized enrichment score |NES| > 1.5.

Gene		MxS ^1^		AxS
Symbol	LogFC	FDR	LogFC	FDR
*Tryptophan metabolism pathway*				
*IDO1*			2.0	5 × 10^−3^
*Ether lipid metabolism pathway*				
*PLD1*	−0.7	3 × 10^−2^		
*LOC110259864*	1. 7	4 × 10^−3^		
*Immune System*				
*CCL2*			1.6	2 × 10^−3^
*CCR1*	2.0	1 × 10^−5^		
*CXCL10*			1.7	1 × 10^−5^
*CXCL9*	−2.2	1 × 10^−5^		
*Cellular Processes*				
*CD14*	1.4	1 × 10^−5^		
*CSF2RB*	1.6	1 × 10^−5^		

^1^ MxS = morphine-by-sex interaction effect; AxS = maternal immune activation (MIA)-by-sex interaction effect where LogFC > 0 (LogFC < 0) denotes gene over-expression (under-expression) in morphine-treated or MIA-exposed females relative to groups differing in sex, morphine treatment or MIA levels.

## Data Availability

The raw and normalized gene expression levels are available in the National Center for Biotechnology Information (NCBI) Gene Expression Omnibus (GEO) database, series identifier GSE209907.

## Supplementary Materials

The following supporting information can be downloaded at: https://www.mdpi.com/article/10.3390/genes13081371/s1, Supplemental Table S1: Log (fold change), False Discovery Rate-adjusted *p*-value (FDR), and raw *p*-values of all genes that presented a significant (FDR *p*-value *<* 0.05 and |log2 (fold change between pig groups)| > 1.2) main effect of maternal immune activation; Supplemental Table S2: Log (fold change), False Discovery Rate-adjusted *p*-value (FDR), and raw *p*-values of all genes that presented a significant (FDR *p*-value < 0.05 and |log2 (fold change between pig groups)| > 1.2) main effect of weaning; Supplemental Table S3: Log (fold change), False Discovery Rate-adjusted *p*-value (FDR), and raw *p*-values of all genes that presented a significant (FDR *p*-value *<* 0.05 and |log2 (fold change between pig groups)| > 1.2) main effect of sex; Supplemental Figure S1: Complete gene networks for the cellular adhesion molecules pathway. Nodes = genes; edges = STRING database connections; node color = differential expression between groups characterized by opioid (M = morphine or S = saline) followed by MIA exposure (C = control or P = viral MIA) from red (under-expressed in first vs. second group, to yellow, and green (over-expressed in first versus second group. Networks: MC vs. SC = morphine-treated Control vs. saline-treated Control; MP vs. MC = morphine-treated MIA vs. morphine-treated Control; MC vs. SP = morphine-treated Control vs. saline-treated MIA; MP vs. SC = morphine-treated MIA vs. saline-treated Control; SP vs. SC = saline-treated MIA vs. saline-treated Control; and MP vs. SP = morphine-treated MIA vs. saline-treated MIA. Supplemental Figure S2. Complete gene networks for the long-term depression pathway. Nodes = genes; edges = STRING database connections; node color = differential expression between groups characterized by opioid (M = morphine or S = saline) followed by MIA exposure (C = control or P = viral MIA) from red (under-expressed in first vs. second group, to yellow, and green (over-expressed in first versus second group. Networks: MC vs. SC = morphine-treated Control vs. saline-treated Control; MP vs. MC = morphine-treated MIA vs. morphine-treated Control; MC vs. SP = morphine-treated Control vs. saline-treated MIA; MP vs. SC = morphine-treated MIA vs. saline-treated Control; SP vs. SC = saline-treated MIA vs. saline-treated Control; and MP vs. SP = morphine-treated MIA vs. saline-treated MIA.

## Abbreviations

ASD	Autism spectrum disorder
FDR	False Discovery Rate
GSEA	Gene Set Enrichment Analysis
KEGG	Kyoto Encyclopedia of Genes and Genomes
LFC	log2(fold change)
LPS	Lipopolysaccharide
MIA	Maternal immune activation
NES	Normalized enrichment score
Poly(I:C)	Polyinosinic-polycytidylic acid
SSD	Schizophrenia spectrum disorder
MC vs. SC	Morphine-treated Control vs. saline-treated Control
MP vs. MC	Morphine-treated MIA vs. morphine-treated Control
MC vs. SP	Morphine-treated Control vs. saline-treated MIA
MP vs. SC	Morphine-treated MIA vs. saline-treated Control
SP vs. SC	Saline-treated MIA vs. saline-treated Control
MP vs. SP	Morphine-treated MIA vs. saline-treated MIA
