# Supplementary material for: Prefrontal Cortex Response to Prenatal Insult and Postnatal Opioid Exposure
Source: Genes (Basel). 2022 Jul 30;13(8):1371. doi: 10.3390/genes13081371 (PMC9407090; doi:10.3390/genes13081371)
Supplement: Supplementary file 1 [file genes-13-01371-s001.zip › genes-1820295-supplementary.pdf]

Supplemental Tables and Figures

Supplementary Table S1. Log2(fold change) (FC) and False Discovery Rat × 10-adjusted P-value (FDR) of genes in the prefrontal cortex that presented at least one significant (FDR P-value < 0.05, and a |log2(fold change)| > 1.2) effect for the main effects and interactions (x) between morphine (M), maternal immune activation (A), and sex (S).

	A x M		A		M		AxS		MxS		S	
Symbol	FC	FDR	FC	FDR	FC	FDR	FC	FDR	FC	FDR	FC	FDR
ADGRE1			7.7	1×10^{-10}					2.9	1×10^{-8}		
ADGRG3							-1.8	2×10^{-3}				
AMBN			1.8	1×10^{-10}								
AQP1									-1.2	1×10^{-3}		
ARG1	-1.6	1×10^{-10}					2.2	7×10^{-5}	2.5	1×10^{-10}		
ATF3			1.3	1×10^{-10}								
B2M			1.2	1×10^{-10}								
BATF2			2.3	1×10^{-10}								
BCL2L14			1.9	1×10^{-10}								
BST2			1.6	1×10^{-10}								
BTNL9			1.2	1×10^{-10}								
C1H15orf48									1.5	1×10^{-3}		
C2			2.5	1×10^{-10}								
C4BPA-2			3.6	1×10^{-10}	-3.1	1×10^{-10}			-2.4	1×10^{-3}		
C7	1.4	1×10^{-10}										
CCL2			2.1	1×10^{-10}	-1.4	1×10^{-10}	1.6	2×10^{-3}				
CCL4			1.7	1×10^{-10}	-1.4	1×10^{-10}						
CCL5			2.9	1×10^{-10}								
CCL8			1.3	1×10^{-10}								
CCR1									1.9	1×10^{-10}		
CCR2			2.1	1×10^{-10}								
CD163			3.0	1×10^{-10}					2.1	1×10^{-10}		
CD2			3.2	1×10^{-10}								
CD274			1.4	1×10^{-10}								
CD300LF			1.3	1×10^{-10}								
CD3E			2.5	1×10^{-10}								
CD3G			3.0	1×10^{-10}								
CD48			1.7	1×10^{-10}								
CD52			2.5	1×10^{-10}			2.2	3×10^{-3}				
CD5L			1.2	1×10^{-10}					-2.3	5×10^{-6}		
CD6			1.9	1×10^{-10}								
CD74			2.6	1×10^{-10}					-1.6	1×10^{-10}		
CHI3L1									1.7	7×10^{-5}		
CRABP1			-1.5	1×10^{-10}								
CRABP2	1.9	1×10^{-10}	-2.4	1×10^{-10}	-1.9	1×10^{-10}			-1.3	5×10^{-2}		
CSF2RB									1.6	4×10^{-6}		
CSF3R	-1.8	1×10^{-10}			-1.2	1×10^{-10}	1.7	1×10^{-2}	2.4	3×10^{-9}		
CTSS			1.4	1×10^{-10}								

CTSW			2.3	1×10^{-10}								
CXCL10			4.7	1×10^{-10}			1.7	4×10^{-8}				
CXCL11			4.9	1×10^{-10}								
CXCL9			5.5	1×10^{-10}					-2.2	5×10^{-6}		
CYTIP			1.8	1×10^{-10}								
DDX60			2.6	1×10^{-10}					-1.4	7×10^{-8}		
DHX58			2.0	1×10^{-10}								
DNTT			1.4	1×10^{-10}								
DSP	1.3	1×10^{-10}	-1.5	1×10^{-10}	-1.3	1×10^{-10}						
EIF1AY							-5.0	1×10^{-10}	6.8	1×10^{-10}	-8.9	1 × 10-10
EMILIN2									1.7	5×10^{-8}		
EOMES			1.5	1×10^{-10}								
EPSTI1			2.0	1×10^{-10}								
ETV7			1.2	1×10^{-10}								
F13A1							1.4	1×10^{-6}	2.2	1×10^{-10}		
FAM167B			2.0	1×10^{-10}					1.5	3×10^{-2}		
FAM26F			1.4	1×10^{-10}								
FCGR2B			2.3	1×10^{-10}								
FCN1			2.4	1×10^{-10}	-1.6	1×10^{-10}			3.4	1×10^{-10}		
GBP1			3.0	1×10^{-10}			1.6	1×10^{-10}	-1.2	1×10^{-10}		
GDPD1	-1.6	1×10^{-10}	2.0	1×10^{-10}								
GNLY			2.9	1×10^{-10}	-1.2	1×10^{-10}						
GPR84	-2.0	1×10^{-10}	2.4	1×10^{-10}	-1.8	1×10^{-10}	2.4	7×10^{-6}	3.2	1×10^{-10}	-1.3	2×10^{-8}
GVIN1			2.2	1×10^{-10}								
GZMA-2			4.5	1×10^{-10}								
GZMB			4.3	1×10^{-10}	-1.5	2×10^{-3}						
HERC5			2.5	1×10^{-10}								
HERC6			1.4	1×10^{-10}								
IDO1					-2.1	1×10^{-10}	2.0	5×10^{-3}				
IER3									1.9	2×10^{-6}		
IFI44			1.7	1×10^{-10}								
IFI44L			1.6	1×10^{-10}								
IFI6			2.0	1×10^{-10}	-1.5	1×10^{-10}						
IFIH1			1.6	1×10^{-10}								
IFIT1			1.8	1×10^{-10}								
IFIT3			1.7	1×10^{-10}								
IFITM1			2.4	1×10^{-10}								
IFITM3			1.4	1×10^{-10}								
IL15RA			1.4	1×10^{-10}								
IL2RB			2.3	1×10^{-10}								
ISG12(A)			1.8	1×10^{-10}	-1.3	1×10^{-10}						
ISG15			1.4	1×10^{-10}								
ISG20			3.6	1×10^{-10}								
ITGB7			1.9	1×10^{-10}								
JCHAIN			1.3	1×10^{-10}								

KLRD1			1.9	1×10^{-10}								
KLRK1			3.6	1×10^{-10}								
KMO			1.3	1×10^{-10}								
KRT19	1.7	1×10^{-10}	-1.7	1×10^{-10}								
LCK			3.1	1×10^{-10}	-1.6	1×10^{-10}						
LGALS3BP			1.6	1×10^{-10}								
LGALS9			1.8	1×10^{-10}								
LOC100038329			4.7	1×10^{-10}					3.1	1×10^{-10}		
LOC100125542			5.2	1×10^{-10}					1.9	3×10^{-8}		
LOC100152327			4.6	1×10^{-10}					2.2	1×10^{-10}		
LOC100153139			2.9	1×10^{-10}	-1.2	4×10^{-8}						
LOC100154053	-1.2	1×10^{-10}	1.8	1×10^{-10}			1.8	3×10^{-2}				
LOC100155195			2.5	1×10^{-10}			1.2	1×10^{-4}				
LOC100156073			1.5	1×10^{-10}								
LOC100158069			1.9	1×10^{-10}								
LOC100513601									-2.4	1×10^{-10}		
LOC100514479											1.4	3×10^{-8}
LOC100514951			1.4	1×10^{-10}								
LOC100516039			1.2	1×10^{-10}								
LOC100516587											1.6	1×10^{-10}
LOC100519082			1.7	1×10^{-10}	-1.3	1×10^{-10}						
LOC100519098			1.5	1×10^{-10}								
LOC100519314			1.5	1×10^{-10}								
LOC100520491			1.3	1×10^{-10}								
LOC100523310			1.6	1×10^{-10}					-1.2	4×10^{-2}		
LOC100524999			1.6	1×10^{-10}					-1.3	4×10^{-3}		
LOC100525452									1.7	1×10^{-3}		
LOC100525692									-5.9	1×10^{-10}		
LOC100526118							-1.7	2×10^{-3}				
LOC100621559			3.0	1×10^{-10}								
LOC100623670	-2.4	2×10^{-6}	6.2	1×10^{-10}					1.8	1×10^{-3}		
LOC100624149							-4.5	1×10^{-10}	7.4	1×10^{-10}	-8.6	1×10^{-10}
LOC100624329											-3.2	1×10^{-10}
LOC100624590							-5.7	1×10^{-10}	8.3	1×10^{-10}	-9.1	1×10^{-10}
LOC100624648							3.6	1×10^{-10}	-1.6	1×10^{-10}	3.7	1×10^{-10}
LOC100624650							1.9	2×10^{-7}				
LOC100625207							-5.8	1×10^{-10}	7.6	1×10^{-10}	-8.8	1×10^{-10}
LOC100626247			1.5	1×10^{-10}								
LOC100737113									1.7	1×10^{-3}		
LOC100737436	1.6	1×10^{-10}	-1.6	1×10^{-10}			-3.7	1×10^{-10}	-3.9	1×10^{-10}		
LOC100739325			2.1	1×10^{-10}								
LOC102160313			2.5	1×10^{-10}								
LOC102162336			1.6	1×10^{-10}								
LOC102163798									-1.2	5×10^{-3}		
LOC102164141	-1.3	1×10^{-10}	1.6	1×10^{-10}								

LOC106504234			3.5	1×10^{-10}			2.1	1×10^{-2}				
LOC106504372			3.9	1×10^{-10}					$^{-2}$.5	1×10^{-10}		
LOC106504562	-1.2	1×10^{-10}										
LOC106505355							2.3	2×10^{-3}	1.9	6×10^{-4}		
LOC110255229			-1.4	1×10^{-10}								
LOC110255257							-6.5	1×10^{-10}	6.8	1×10^{-10}	-9.1	1×10^{-10}
LOC110255320							-5.5	1×10^{-10}	7.5	1×10^{-10}	-8.4	1×10^{-10}
LOC110255360			1.8	1×10^{-10}								
LOC110255361			2.5	1×10^{-10}								
LOC110255657	1.7	1×10^{-10}					$^{-2}$.1	8×10^{-6}				
LOC110256043			5.1	1×10^{-10}					-1.8	8×10^{-8}		
LOC110256714			6.4	1×10^{-10}					2.4	3×10^{-8}		
LOC110256816							1.9	3×10^{-2}				
LOC110257240			3.0	1×10^{-10}								
LOC110257381	1.4	1×10^{-10}										
LOC110257883							-4.5	1×10^{-7}	6.9	1×10^{-10}	-8.0	1×10^{-10}
LOC110257894							-2.5	1×10^{-5}	3.2	1×10^{-10}	-5.1	1×10^{-10}
LOC110257896							-3.0	4×10^{-4}	4.2	1×10^{-10}	-5.8	1×10^{-10}
LOC110257905							-3.4	1×10^{-10}	6.8	1×10^{-10}	-8.2	1×10^{-10}
LOC110257919			1.8	1×10^{-10}								
LOC110258060											1.2	1×10^{-10}
LOC110258195							-1.8	2×10^{-2}				
LOC110258326			4.9	1×10^{-10}			-2.4	1×10^{-10}				
LOC110258673	-1.7	5×10^{-2}	6.0	1×10^{-10}					2.1	8×10^{-5}		
LOC110258921			1.5	1×10^{-10}								
LOC110259088			1.5	1×10^{-10}								
LOC110259530			1.2	1×10^{-9}								
LOC110259864									1.7	4×10^{-3}		
LOC110259983			6.5	1×10^{-10}					2.3	4×10^{-7}		
LOC110260608			-1.7	1×10^{-10}	1.4	1×10^{-10}						
LOC110260829	1.6	1×10^{-10}					-1.5	1×10^{-8}				
LOC396781			5.4	1×10^{-10}			-1.2	3×10^{-4}	2.3	1×10^{-10}		
LOX			-1.9	1×10^{-10}								
LY86			2.4	1×10^{-10}								
LY9			2.0	1×10^{-10}								
LYZ	-1.8	1×10^{-10}	3.0	1×10^{-10}	$^{-2}$.1	1×10^{-10}			3.5	1×10^{-10}		
MARCO			5.6	1×10^{-10}								
MEFV									1.9	3×10^{-5}		
MX2			2.9	1×10^{-10}								
NLRC5			1.5	1×10^{-10}								
NPSR1			-1.6	1×10^{-10}					-3.6	1×10^{-10}		
OAS1							1.3	1×10^{-3}	-2.0	1×10^{-10}		
OASL	-1.2	7×10^{-4}	4.7	1×10^{-10}					-1.3	4×10^{-2}		
OLR1			1.7	1×10^{-10}	-1.3	1×10^{-10}						
PARP12			1.2	1×10^{-10}								

PARP14			2.0	1×10^{-10}								
PARP9			1.3	1×10^{-10}								
PI15	1.2	1×10^{-10}			-1.6	1×10^{-10}			-1.4	1×10^{-2}		
PILRA			1.6	1×10^{-10}					1.6	8×10^{-3}		
PLAC8			2.4	1×10^{-10}			1.6	3×10^{-4}	-1.4	2×10^{-5}		
PNPLA4-2									-1.7	1×10^{-4}		
POSTN							1.5	3×10^{-3}				
PRF1			2.7	1×10^{-10}								
PSMB8			1.6	1×10^{-10}								
PSMB9			1.8	1×10^{-10}								
PTPN7			1.5	1×10^{-10}								
PTPRC			1.5	1×10^{-10}								
PYURF	5.6	1×10^{-10}	5.6	1×10^{-10}	-5.4	1×10^{-10}	8.2	1×10^{-10}	-2.8	1×10^{-10}	4.2	1×10^{-10}
RHBDF2			1.6	1×10^{-10}								
RSAD2			4.5	1×10^{-10}			1.2	4×10^{-4}				
RUNX3			1.9	1×10^{-10}								
S100A4					-1.2	1×10^{-10}						
SAA3	-2.7	1×10^{-7}	6.2	1×10^{-10}	-2.5	5×10^{-5}			2.8	1×10^{-10}		
SAMSN1			1.4	1×10^{-10}								
SDS									1.8	1×10^{-6}		
SELE			3.0	1×10^{-10}	-1.5	1×10^{-10}			2.9	1×10^{-10}		
SELL									1.7	5×10^{-5}		
SERPINE1			1.2	1×10^{-10}					2.5	1×10^{-10}		
SFRP5	1.3	1×10^{-10}			-1.3	1×10^{-10}			-1.4	4×10^{-2}		
SIGLEC1			1.8	1×10^{-10}								
SIGLEC14												
SLA-2			1.7	1×10^{-10}								
SLA^{-3}			1.3	1×10^{-10}								
SLA^{-5}	-1.3	1×10^{-10}	1.7	1×10^{-10}								
SLA-DMA			1.6	1×10^{-10}								
SLA-DOA			3.4	1×10^{-10}					-2.3	4×10^{-5}		
SLA-DQA1			3.2	1×10^{-10}	-1.2	1×10^{-10}	1.3	7×10^{-4}	-1.4	6×10^{-8}		
SLA-DQB1			2.6	1×10^{-10}					-1.5	1×10^{-10}		
SLA-DRA			3.8	1×10^{-10}	-1.3	1×10^{-10}			-1.9	1×10^{-10}		
SLA-DRB1			2.3	1×10^{-10}			1.5	3×10^{-4}	-1.2	2×10^{-4}		
SLC13A4	1.3	1×10^{-10}										
SLC5A5	1.8	1×10^{-10}	-1.7	1×10^{-10}					-1.5	2×10^{-3}		
SOCS3			1.5	1×10^{-10}					2.6	1×10^{-10}		
SPN			1.3	1×10^{-10}								
SRGN			1.3	1×10^{-10}					1.5	1×10^{-10}		
STX11			2.4	1×10^{-10}								
TAP1			1.5	1×10^{-10}								
TAP2			1.2	1×10^{-10}								
TBX21			2.7	1×10^{-10}	-1.4	5×10^{-6}						
TGM1									1.7	1×10^{-3}		

THBS1										
TIMP1					-1.3	1×10^{-10}	1.2	7×10^{-4}	1.5	1×10^{-10}
TMEM156			1.2	1×10^{-10}						
TNMD	1.4	1×10^{-10}	-1.3	1×10^{-10}	-1.6	1×10^{-10}			-1.4	2×10^{-2}
TTR									2.1	2×10^{-9}
UBA7			1.3	1×10^{-10}						
UBD			4.1	1×10^{-10}			1.7	1×10^{-2}		
UNC93B1				1.4	1×10^{-10}					
USP18				2.0	1×10^{-10}					
XAF1				1.7	1×10^{-10}					
ZBP1				2.1	1×10^{-10}					

Supplemental Table S2. Log2(fold change) of pairwise contrasts between treatment groups for genes in the cell adhesion molecules pathway.

Gene	MC vs SC[1]	MP vs MP	MC vs SP	MP vs SC	SP vs SC	MP vs SP
CD2	-0.01	2.29	-4.21	2.29	4.21	-1.92
CD22	0.49	0.29	-0.85	0.77	1.34	-0.56
CD274	0.24	0.54	-1.92	0.78	2.17	-1.39
CD4	0.03	-0.33	0.09	-0.30	-0.06	-0.23
CD40	-0.24	0.52	-1.07	0.28	0.83	-0.55
CD6	-0.39	1.42	-2.84	1.02	2.44	-1.42
CD80	0.38	-0.01	0.09	0.38	0.29	0.09
CD8A	0.10	0.35	-0.69	0.45	0.79	-0.34
CDH1	-1.86	0.16	-0.53	-1.70	-1.32	-0.38
CDH2	0.36	-0.14	0.02	0.22	0.34	-0.12
CLDN6	-1	0.33	-0.50	-0.67	-0.50	-0.17
CNTN1	0.33	-0.12	0.18	0.21	0.14	0.06
F11R	0.19	0.08	-0.15	0.27	0.34	-0.07
ICAM1	-0.26	0.47	-1.15	0.21	0.88	-0.67
ICAM2	-0.41	0.49	-1.14	0.07	0.73	-0.65
ICAM3	-0.49	0.34	-0.71	-0.15	0.22	-0.38
ITGA4	0.48	0.31	-1.09	0.79	1.57	-0.78
ITGA6	0.19	-0.21	0.13	-0.02	0.06	-0.08
ITGA8	0.34	-0.19	0.07	0.15	0.27	-0.11
ITGAL	0.01	0.20	-0.46	0.22	0.47	-0.26
ITGAM	-0.04	0.39	-0.42	0.35	0.38	-0.03
ITGB2	0.07	0.20	-0.61	0.27	0.68	-0.41
ITGB7	-0.14	1.41	-2.58	1.28	2.45	-1.17
ITGB8	0.55	-0.17	0.35	0.38	0.20	0.18
L1CAM	0.13	-0.07	-0.04	0.06	0.18	-0.12
MADCAM1	-0.44	0.57	-0.80	0.12	0.36	-0.23
NCAM2	0.35	-0.11	0.10	0.23	0.25	-0.01
NEGR1	0.29	-0.12	-0.01	0.17	0.29	-0.12
NFASC	0.49	-0.16	0.36	0.33	0.13	0.21
NLGN1	0.24	-0.11	0.10	0.13	0.14	-0.01
NRCAM	0.29	-0.14	0.12	0.15	0.17	-0.02
NRXN3	0.20	-0.08	-0.02	0.12	0.22	-0.10
NTNG1	0.17	-0.06	-0.15	0.11	0.31	-0.21
PTPRC	0.24	0.91	-1.93	1.15	2.17	-1.02

SDC1	-0.28	-0.01	-0.33	-0.28	0.06	-0.33
SELE	-0.84	2.26	$^{-4}$.50	1.43	3.67	-2.24
SELL	0.05	0.17	-2.07	0.22	2.12	-1.90
SELP	-0.40	1.02	-1.48	0.62	1.08	-0.46
SELPLG	-0.04	0.27	-0.29	0.23	0.25	-0.02
SIGLEC1	-0.19	1.25	-2.45	1.05	2.26	-1.21
SPN	-0.29	1.02	-1.79	0.73	1.50	-0.77
TIGIT	-0.01	0.19	-0.67	0.18	0.66	-0.48
VCAN	0.44	-0.12	0.17	0.33	0.27	0.06

[1]MC = morphin × 10-treated Control group, SC = salin × 10-treated Control group, MP = morphin × 10-treated MIA-exposed group, SP = salin × 10-treated MIA-exposed group.

Supplemental Table S3. Log2(fold change) of pairwise contrasts between treatment groups for genes in the long-term depression pathway.

Gene	MC vs SC[1]	MP vs MP	MC vs SP	MP vs SC	SP vs SC	MP vs SP
BRAF	0.29	-0.08	0.11	0.21	0.19	0.02
CACNA1A	0.26	-0.06	0.06	0.20	0.20	-0.01
GNA13	0.21	-0.05	0.05	0.16	0.16	-0.01
GNAI1	0.28	-0.12	0.06	0.16	0.22	-0.05
GNAQ	0.42	-0.13	0.08	0.29	0.34	-0.05
GRIA1	0.15	-0.07	0.02	0.08	0.13	-0.05
GRIA2	0.46	-0.15	0.07	0.31	0.39	-0.08
GRIA3	0.14	-0.04	-0.01	0.10	0.15	-0.05
GRID2	0.30	-0.12	0.27	0.18	0.03	0.15
GRM1	0.45	-0.11	-0.06	0.33	0.51	-0.17
GUCY1A2	0.53	-0.22	0.14	0.31	0.39	-0.08
IGF1R	0.30	-0.03	0.05	0.27	0.25	0.02
ITPR1	0.26	-0.04	-0.01	0.22	0.27	-0.05
ITPR2	0.07	0.01	0.14	0.07	-0.07	0.14
ITPR3	0.19	-0.06	0.28	0.13	-0.08	0.22
KRAS	0.46	-0.19	0.11	0.27	0.35	-0.08
LYN	0.10	-0.03	-0.05	0.07	0.15	-0.08
MAP2K1	-0.06	0.05	-0.13	-0.02	0.06	-0.08
MAPK1	0.10	-0.05	-0.03	0.05	0.13	-0.08
PLA2G4A	0.05	0.10	-0.36	0.15	0.41	-0.26
PLA2G4B	0.16	0.07	0.07	0.23	0.10	0.13
PLA2G4E	0.21	-0.12	-0.02	0.08	0.22	-0.14
PLCB1	0.19	-0.07	0.02	0.12	0.17	-0.05
PLCB2	0.15	0.03	-0.20	0.17	0.34	-0.17
PPP1R17	0.08	-0.14	0.02	-0.06	0.06	-0.12
PRKCB	0.09	-0.06	-0.02	0.03	0.11	-0.08
PRKG1	0.30	-0.12	0.16	0.18	0.14	0.05
PRKG2	0.35	-0.01	0.32	0.33	0.02	0.31
RYR1	0.52	0.36	-0.04	0.87	0.56	0.32

[1]MC = morphin × 10-treated Control group, SC = salin × 10-treated Control group, MP = morphin × 10-treated MIA-exposed group, SP = salin × 10-treated MIA-exposed group.

CM vs CS

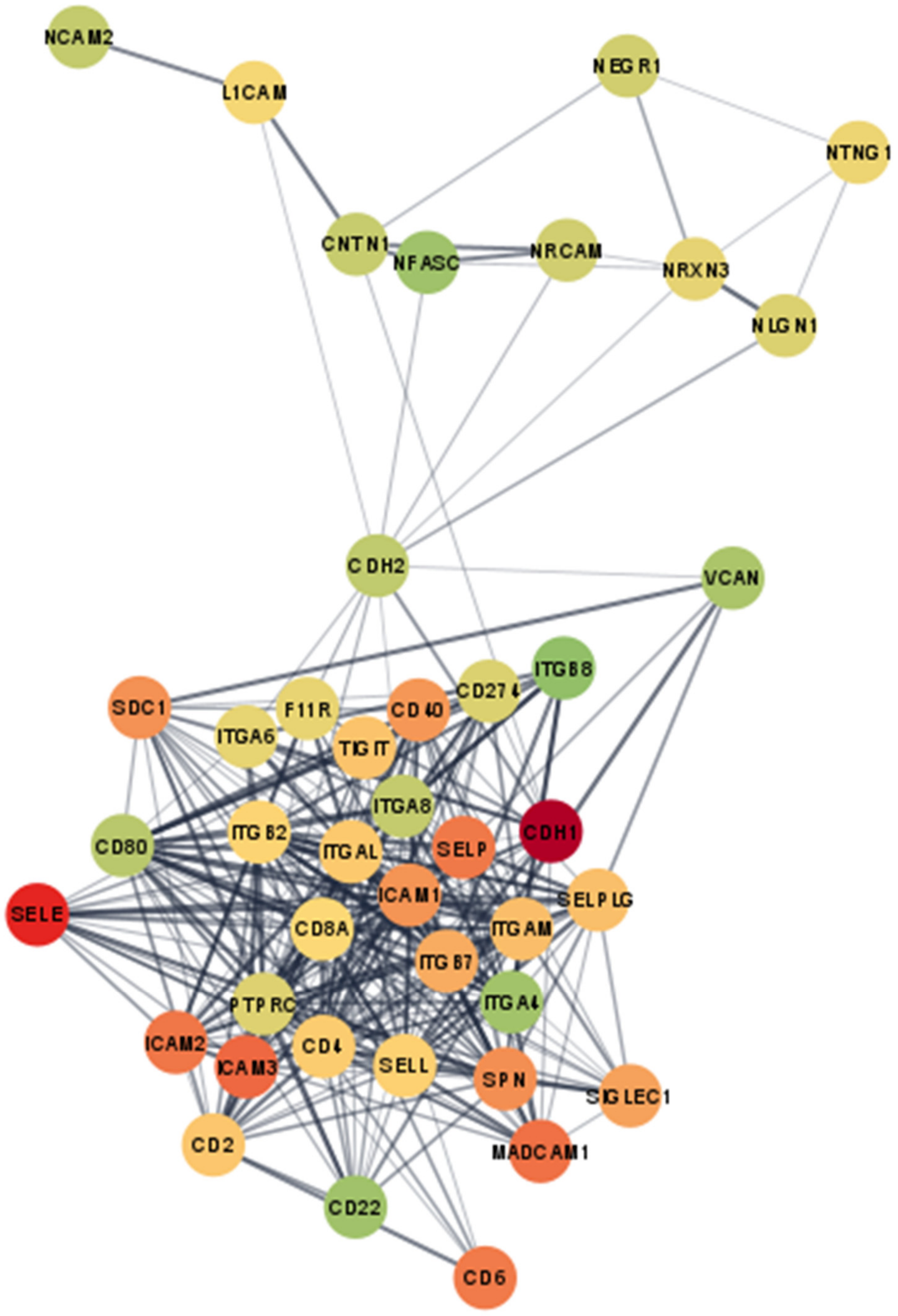

NCAM2
L1CAM
NEGR1
NTNG1
CNTN1
NFASC
NRCAM
NRXN3
NLGN1
CDH2
VCAN
ITGB8
CD274
SDC1
F11R
CD40
ITGA6
TIGIT
ITGA8
CDH1
CD80
ITGB2
ITGAL
SELP
SELE
ICAM1
SELPLG
CD8A
ITGAM
ITGB7
PTPRC
ITGA4
ICAM2
CD4
ICAM3
SELL
SPN
SIGLEC1
CD2
MADCAM1
CD22
CD6

PM vs CM

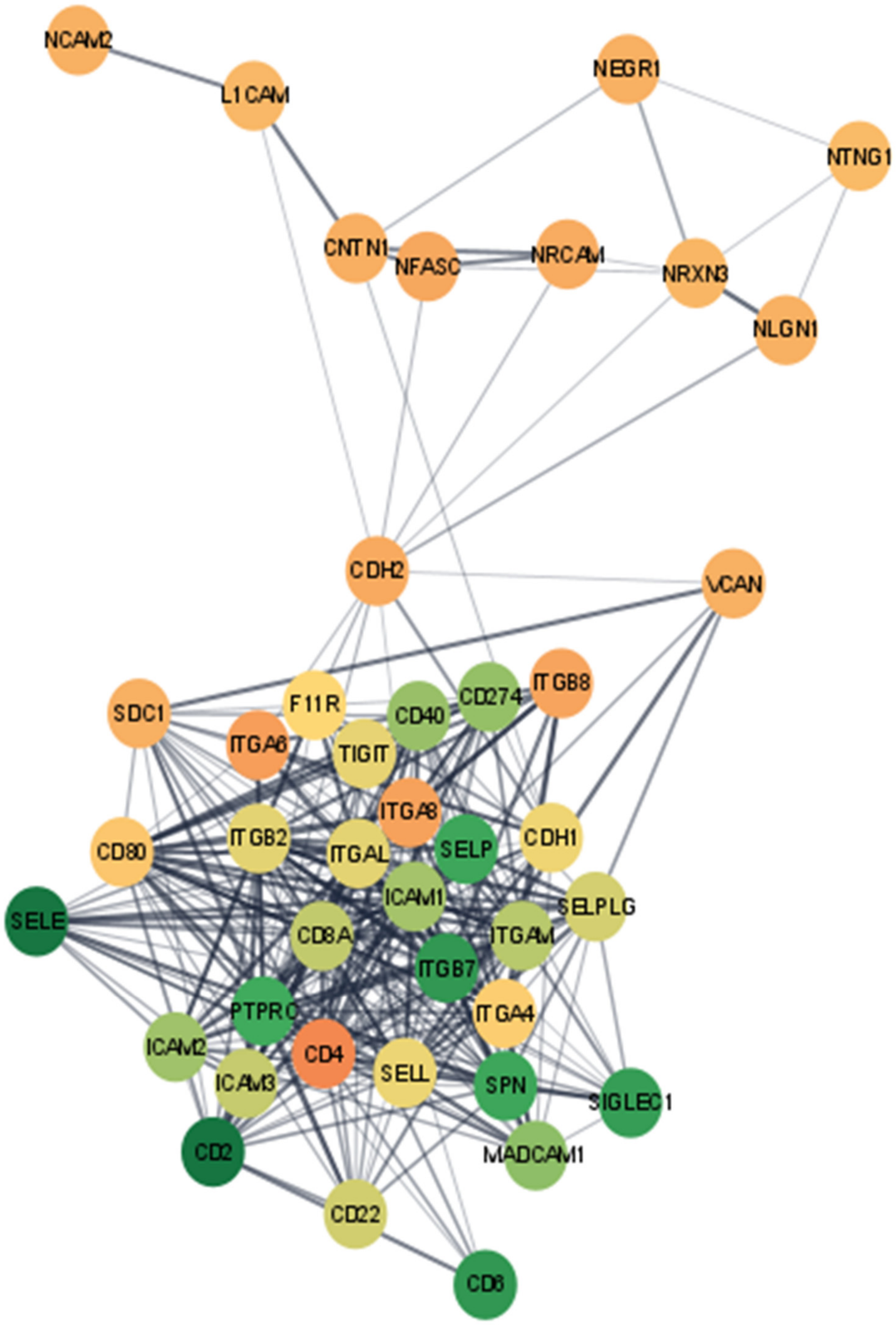
NCAM2
L1CAM
NEGR1
NTNG1
CNTN1
NFASC
NRCAM
NRXN3
NLGN1
CDH2
VCAN
SDC1
F11R
CD40
CD274
ITGB8
ITGA6
TIGIT
ITGA8
CD80
ITGB2
ITGAL
SELP
CDH1
ICAM1
SELPLG
SELE
CD8A
ITGAM
ITGB7
PTPRC
ITGA4
ICAM2
CD4
ICAM3
SELL
SPN
SIGLEC1
MADCAM1
CD2
CD22
CD6

CM vs PS

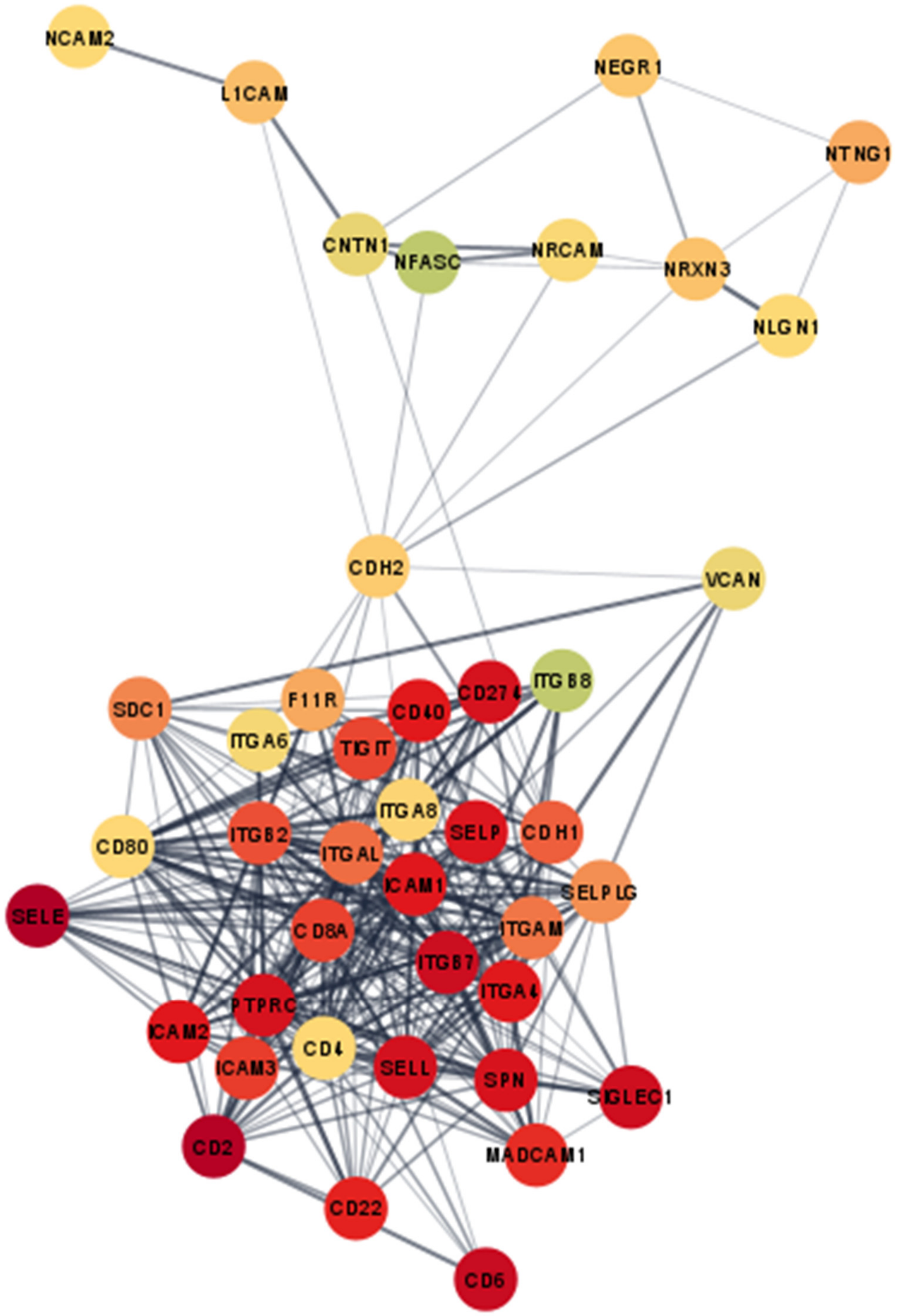
NCAM2
L1CAM
NEGR1
NTNG1
CNTN1
NFASC
NRCAM
NRXN3
NLGN1
CDH2
VCAN
SDC1
F11R
CD274
ITGB8
CD40
ITGA6
TIGIT
ITGA8
CD80
ITGB2
ITGAL
SELP
CDH1
ICAM1
SELPLG
SELE
CD8A
ITGAM
ITGB7
ITGA4
PTPRC
ICAM2
CD4
ICAM3
SELL
SPN
SIGLEC1
CD2
MADCAM1
CD22
CD6

PM vs CS

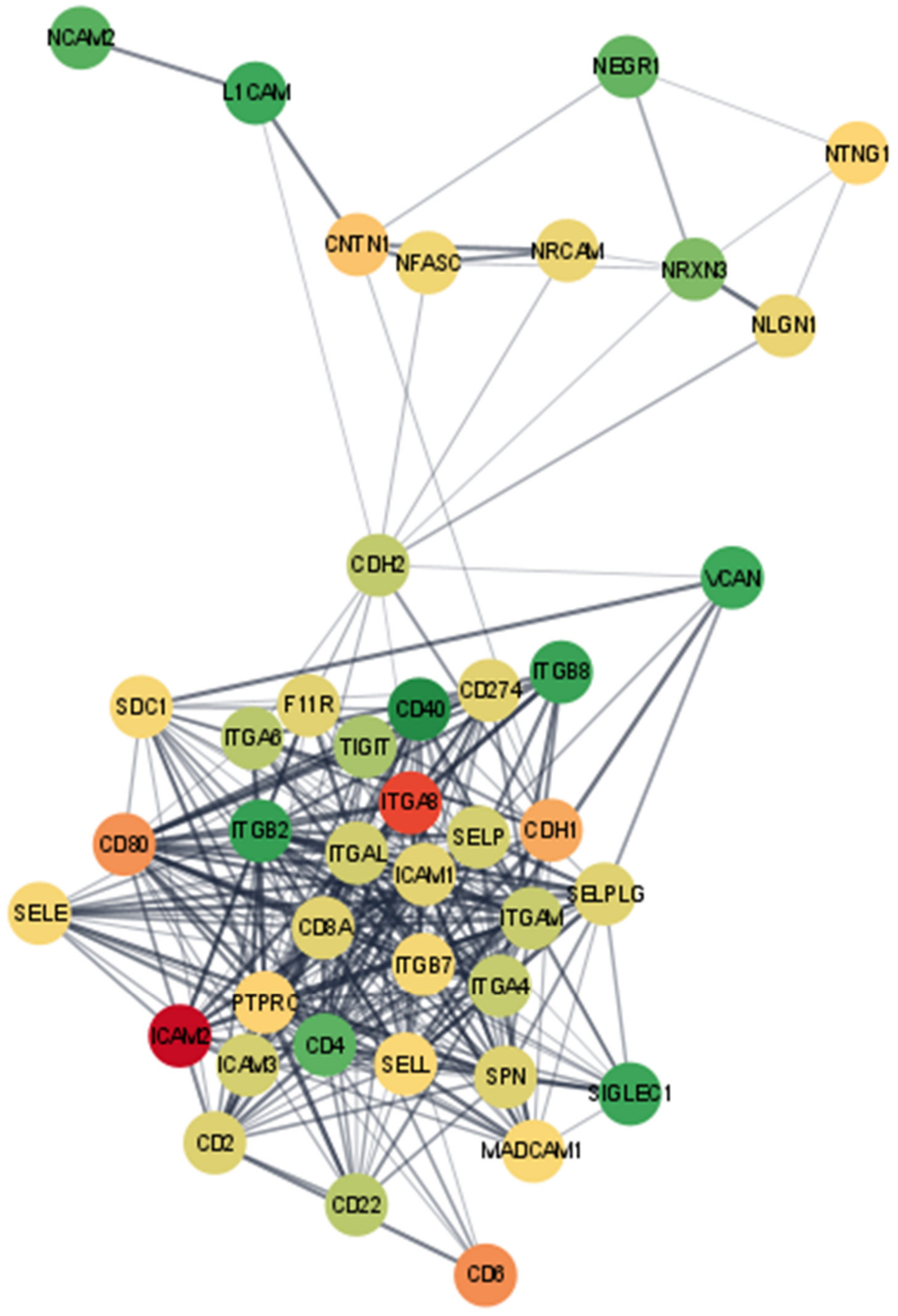
NCAM2
L1CAM
NEGR1
NTNG1
CNTN1
NFASC
NRCAM
NRXN3
NLGN1
CDH2
VCAN
ITGB8
CD274
SDC1
F11R
CD40
ITGA6
TIGIT
ITGA8
CD80
ITGB2
ITGAL
SELP
CDH1
ICAM1
SELPLG
SELE
CD8A
ITGAM
ITGB7
ITGA4
PTPRC
ICAM2
CD4
ICAM3
SELL
SPN
SIGLEC1
MADCAM1
CD2
CD22
CD6

PS vs CS

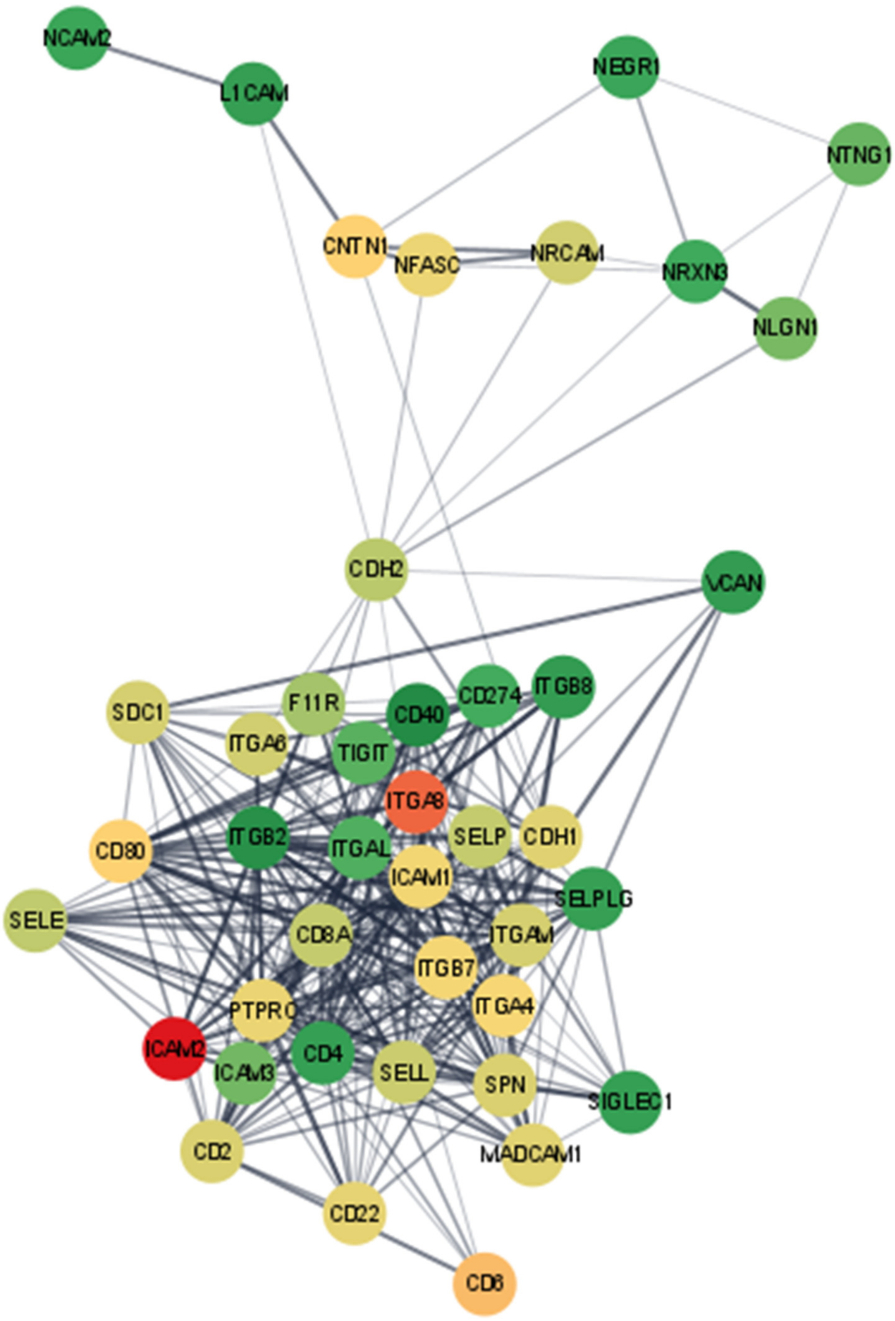

PM vs PS

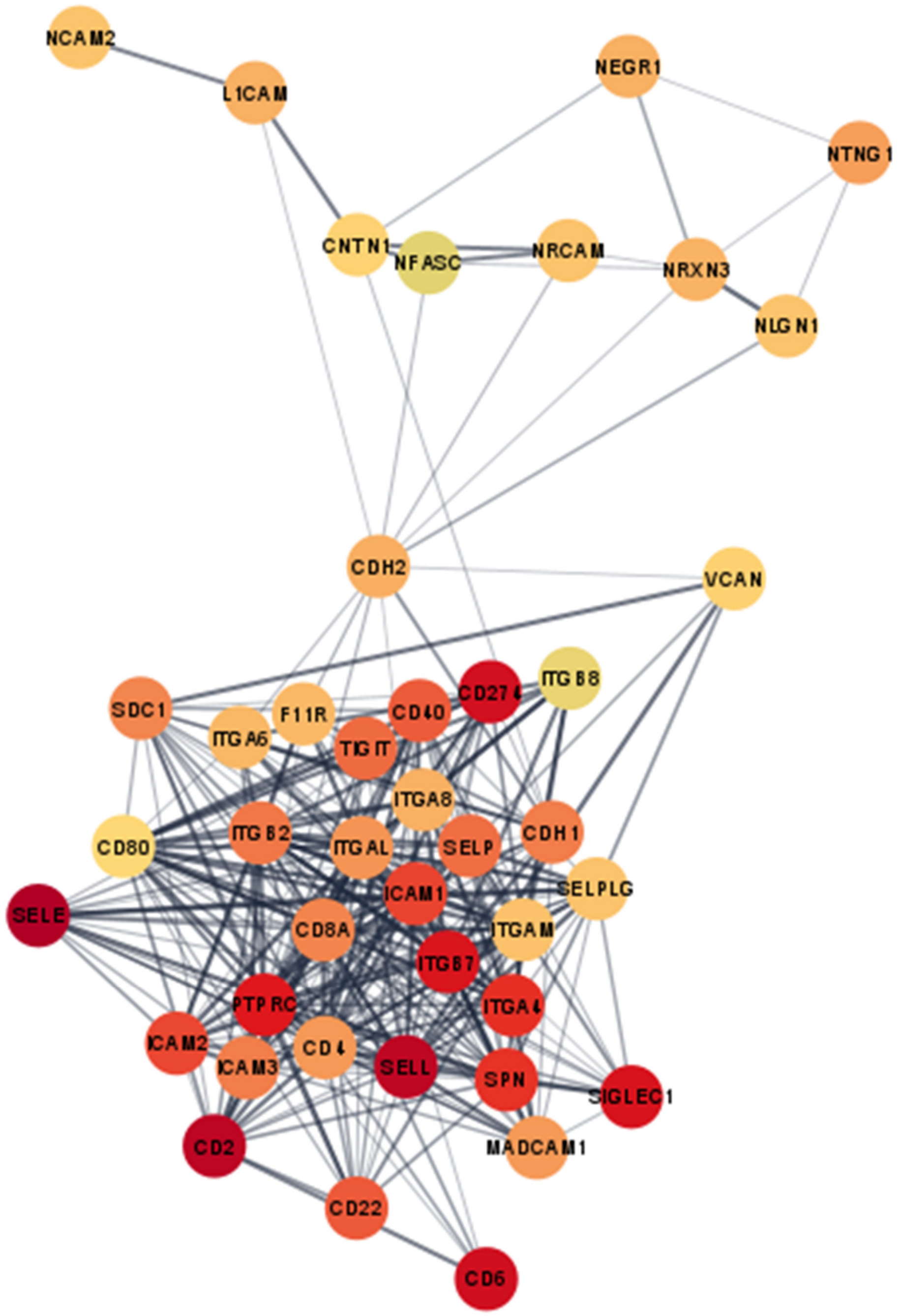

Supplemental Figure S1. Complete gene networks for the cellular adhesion molecules pathway. Nodes = genes; edges = STRING database connections; node color = differential expression between groups characterized by opioid (M=morphine or S=saline) followed by MIA exposure (C=control or P=viral MIA) from red (under-expressed in first vs second group, to yellow, and green (over-expressed in first versus second group. Networks: MC vs SC = morphin × 10-treated Control vs salin × 10-treated Control; MP vs MC = morphin × 10-treated MIA vs morphin × 10-treated Control; MC vs SP = morphin × 10-treated Control vs salin × 10-treated MIA; MP vs SC = morphin × 10-treated MIA vs salin × 10-treated Control; SP vs SC = salin × 10-treated MIA vs salin × 10-treated Control; and MP vs SP = morphin × 10-treated MIA vs salin × 10-treated MIA.

CM vs CS

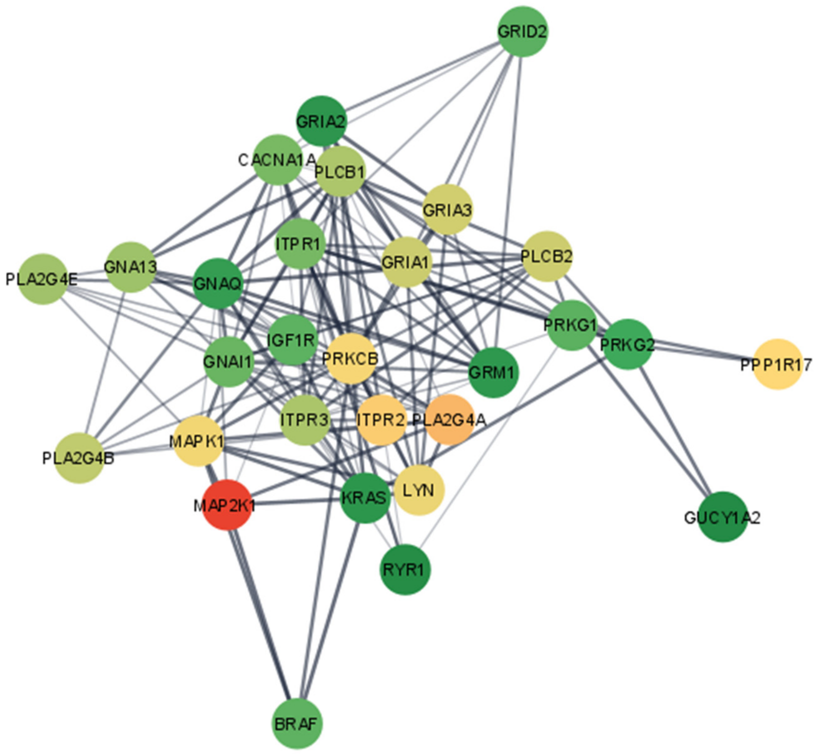

PM vs CM

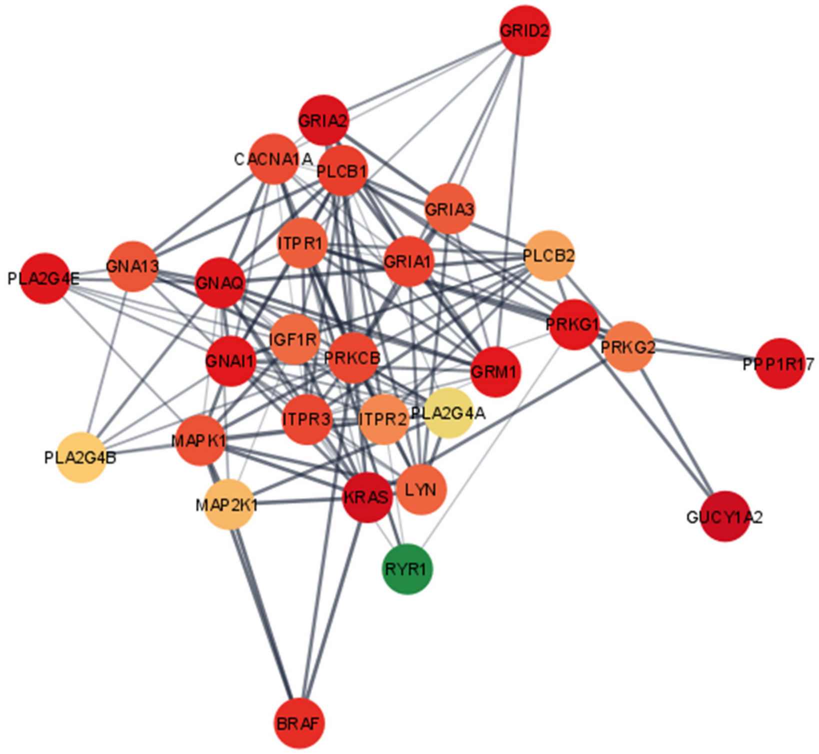

CM vs PS

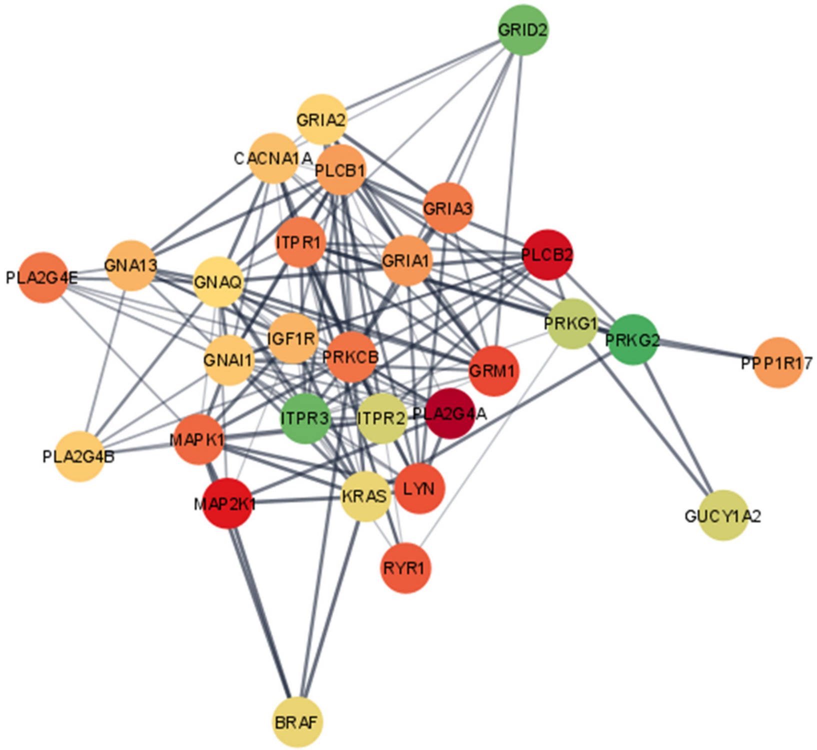

PM vs CS

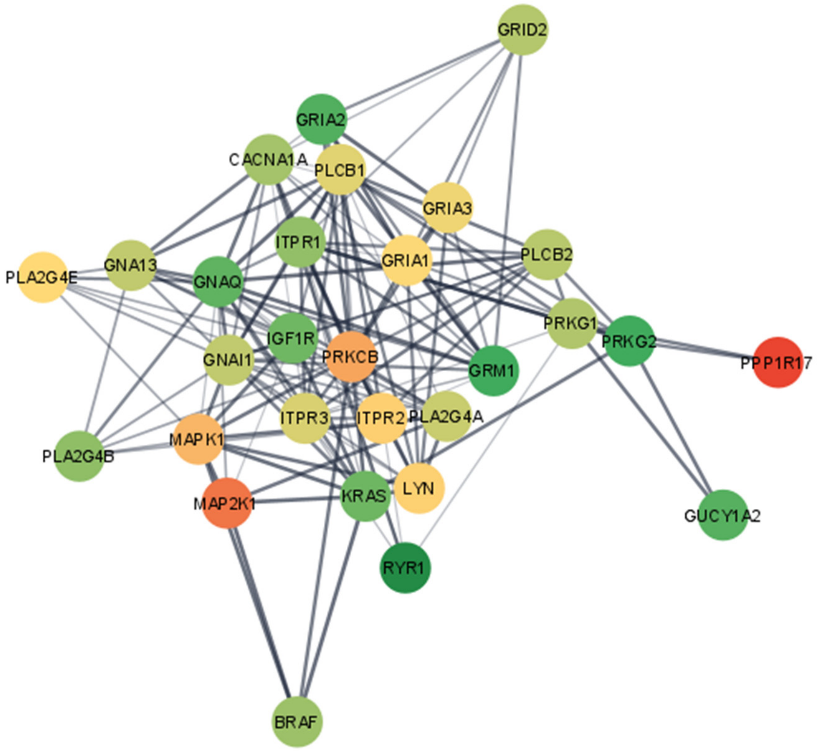

PS vs CS

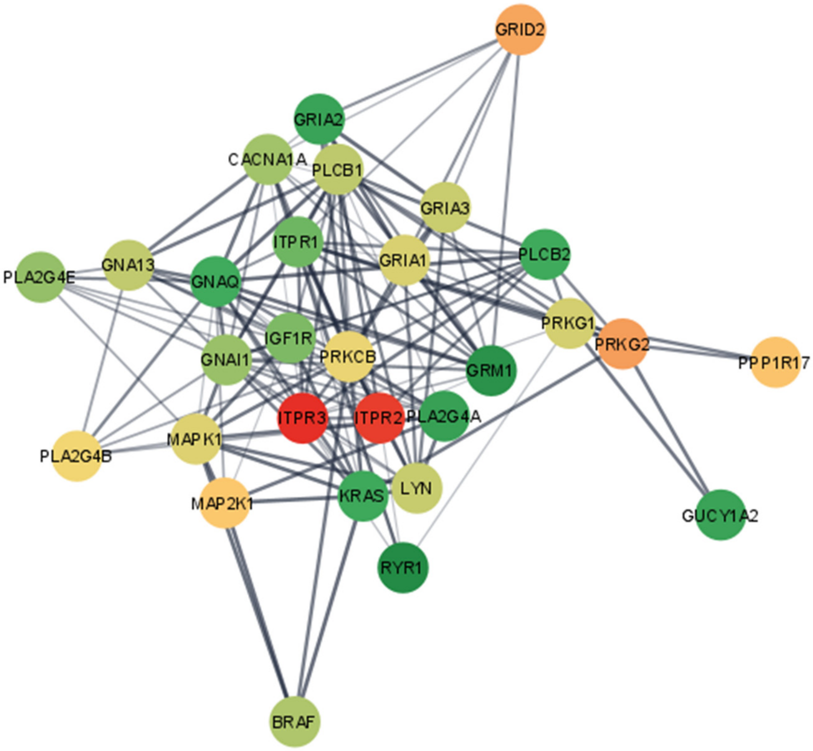

PM vs PS

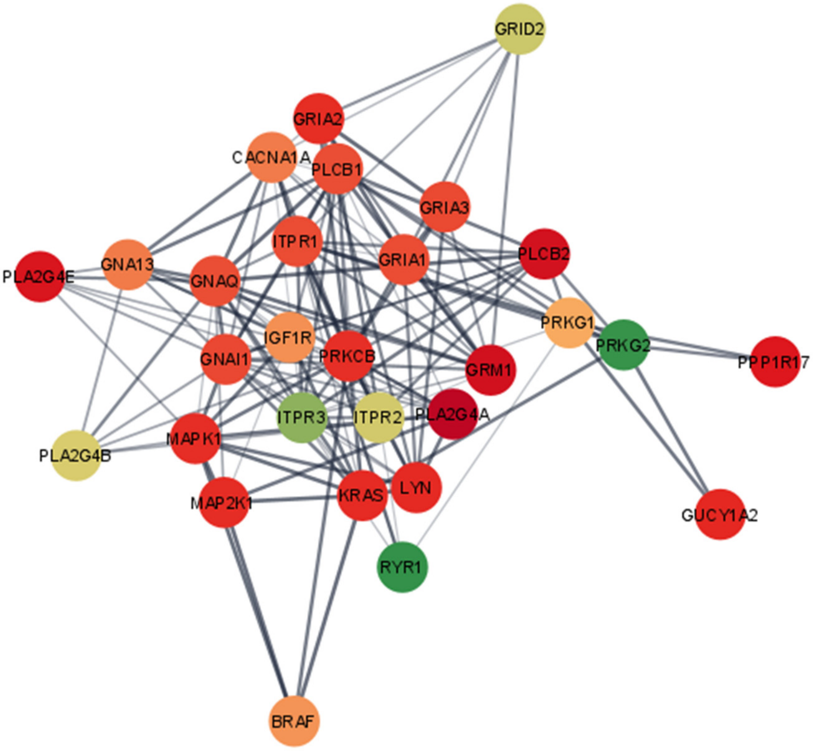

Supplemental Figure S2. Complete gene networks for the long-term depression pathway. Nodes = genes; edges = STRING database connections; node color = differential expression between groups characterized by opioid (M=morphine or

S=saline) followed by MIA exposure (C=control or P=viral MIA) from red (under-expressed in first vs second group, to yellow, and green (over-expressed in first versus second group. Networks: MC vs SC = morphin × 10-treated Control vs salin × 10-treated Control; MP vs MC = morphin × 10-treated MIA vs morphin × 10-treated Control; MC vs SP = morphin × 10-treated Control vs salin × 10-treated MIA; MP vs SC = morphin × 10-treated MIA vs salin × 10-treated Control; SP vs SC = salin × 10-treated MIA vs salin × 10-treated Control; and MP vs SP = morphin × 10-treated MIA vs salin × 10-treated MIA.
